# Supplementary material for: Production-induced seismicity indicates a low risk of strong earthquakes in the Groningen gas field
Source: Nat Commun. 2024 Jan 6;15:329. doi: 10.1038/s41467-023-44485-4 (PMC10771524; doi:10.1038/s41467-023-44485-4)
Supplement: Supplementary file 1 — Supplementary Information [file 41467_2023_44485_MOESM1_ESM.pdf]

Uncertainty estimation of LB-parameters

In the analysis above, we estimated the uncertainty of the LB-parameters based on the co-variance matrices obtained from the curve fitting. Here, we test an alternative approach to assess the uncertainty of these parameters. For this, we randomly selected a certain number of earthquakes (scenario I: 100, scenario II: 600) above the magnitude of completeness from the whole Groningen catalog. Subsequently, we fit the LB-model to this subset of data. This process is repeated 1000 times, with different selections from the catalog. This procedure allows a statistical analysis of the uncertainty of the parameters which is shown in Figure S1. The Figure shows that the uncertainties for all three LB-parameters are significantly higher for the scenario with fewer events, which is an expectable result. The analysis also shows that the uncertainties are significantly higher than those obtained from the covariance matrices. Thus, the drop in the b-value in 2004 might be at least partly explained by a statistical variance. The uncertainties, especially of the b-value, estimated with this method are higher than the temporal variance of the LB-parameters. Thus, it might be also a reasonable assumption to use a time-constant b-value for the whole observation period.

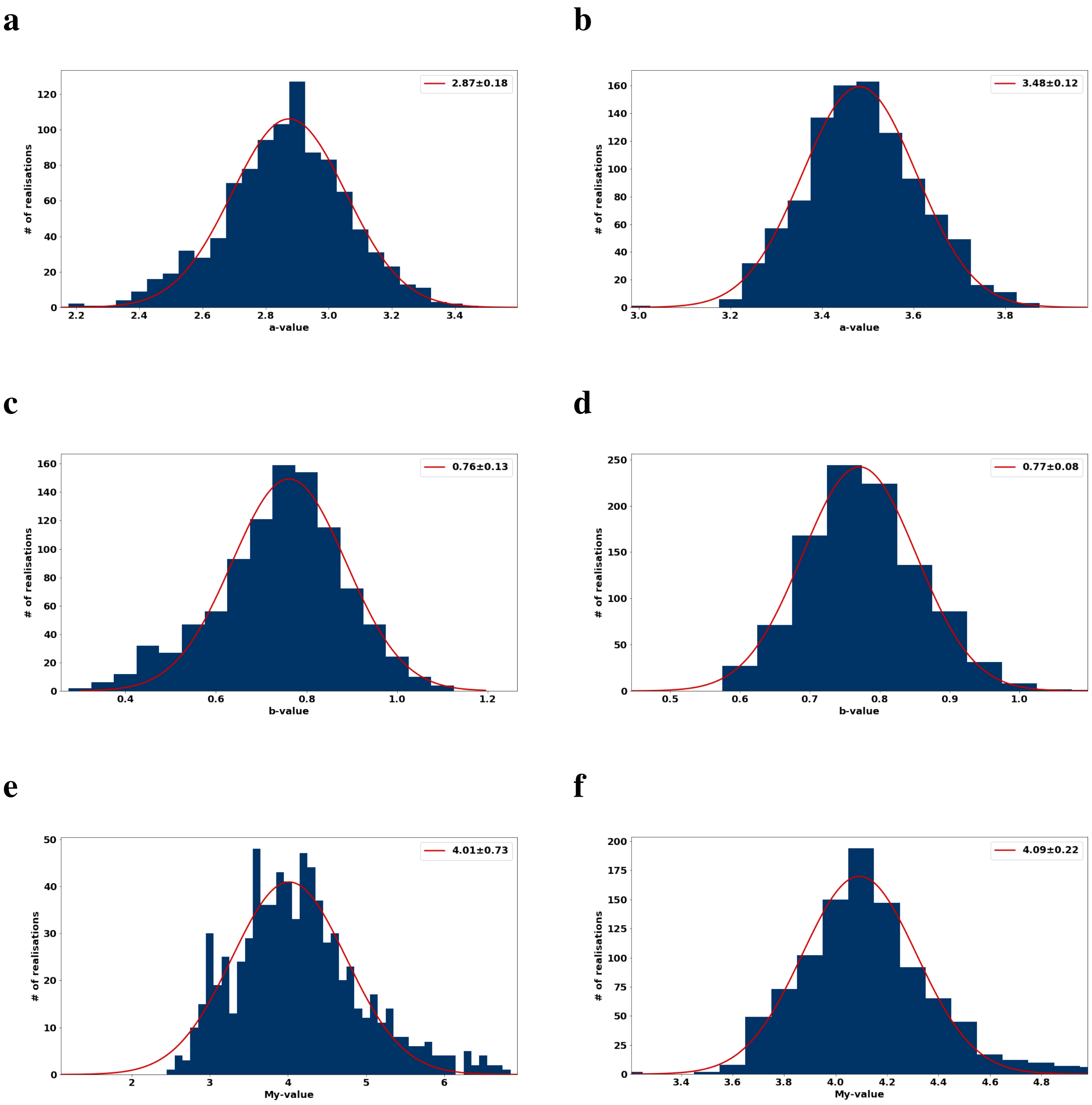

Supplementary Fig. 1: **Uncertainty estimates of the LB-parameters for randomly selected events.** Top: a-value, middle: b-value, bottom: My. Left column: Uncertainty estimation for N=100 events, right column: Uncertainty estimation for N=600 events. The two scenarios correspond approximately to the cumulative observed number of events until the years 2003 and 2019. Uncertainties for the larger event number are significantly smaller, but larger than uncertainties from the covariance-matrices (Figures 2d and 2f)

Exact and Approximated Lower Bound

As discussed in the theory section, fitting Equation 4 to a given frequency magnitude might become problematic, since the b-value and M_Y are coupled in the third term. Thus, we replaced the last term with its Taylor expansion around b equal one (Eq: 5). This approximation is exact for b equal one and deviates from Equation 4 for values different from one. Here, we analyze the magnitudes of these deviations and show that the usage of Equation 5 is a valid assumption also for values significantly different from one. For this, we compute synthetic frequency-magnitude distributions for fixed a- and M_Y values (3.0 and 4.0) and varying b-values (x-axis in Figure S2) using Equation 4. Then we numerically fit Equation 5 to these synthetic distributions and obtain estimates of b- and M_Y values (see Figure S2). The Figure shows, that also for b-values significantly different from one, equation 5 is a reasonable assumption. For b-values smaller than one (i.e the case in Groningen), the approximation slightly overestimates the b-value and underestimates M_Y, which can be also seen in Figure S2b. However, the deviations are within the 1σ-uncertainty, and thus, Equation 5 is a valid assumption.

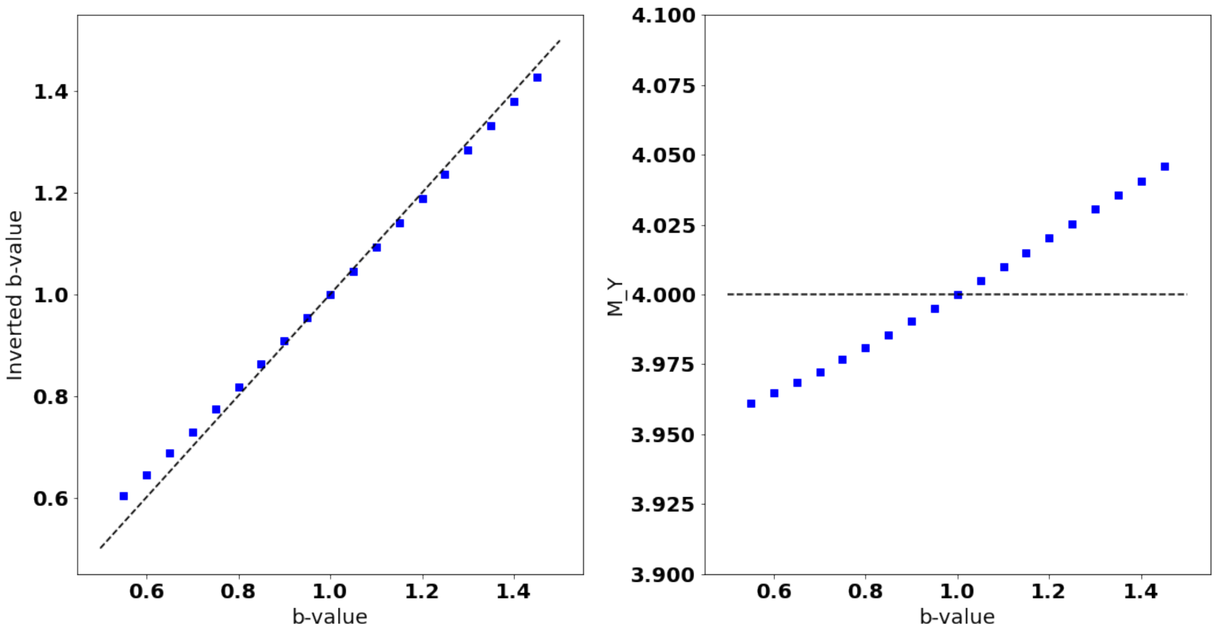

Supplementary Fig. 2: **Uncertainty estimates for b and M_Y comparing Equations 4 and 5. a** Inverted b-value and **b** inverted M_Y of a frequency magnitude created by the exact LB-Equation (Eq: 4) using varying b-values (x-axis) and fitted by the approximated LB-Equation (Eq: 5). Equation 5 is a good approximation of the exact equation, the differences are smaller than the uncertainty. The computed b- and M_Y values from the FM-distribution (Figure 2c) show the same trend.

Worst and Best-Case Probabilities based on LB and GR

The seismogenic index model was initially derived from a GR-like FM distribution. Likewise, the Worst-Case Exceedance Probability was derived for seismogenic indices derived from GR-statistics. As shown, observed seismicity in Groningen differs from the GR-statistic and is better described by the LB-statistic. Thus, we use a- and b-values from the LB to estimate the seismogenic index and the WCEP above. For comparison, we show here the differences between SI and WCEP derived from GR and LB-statistics. The probabilities as well as the expected number of earthquakes differ significantly between the two methods. Probabilities from GR-statistics are generally smaller. However, probabilities are still large (e.g. more than 60% probability of a $M_w 4.0$ in 2021). This means, that independently from the statistical model, large-magnitude earthquakes should have been triggered, if this would be physically possible. Analogously to the WCEP, it is possible to compute a Best-Case triggering probability, by interchanging the supremum and infimum in equation 8. This scenario corresponds to the case, where the medium would react least critically to the occurring pore-pressure changes. Thereby, it is possible to define a range of probabilities, which is shown by the colored shaded areas in Figure S3 a and b. Even for the best case, probabilities are significant which furthermore supports the theory that triggering seismicity in Groningen is highly improbable.

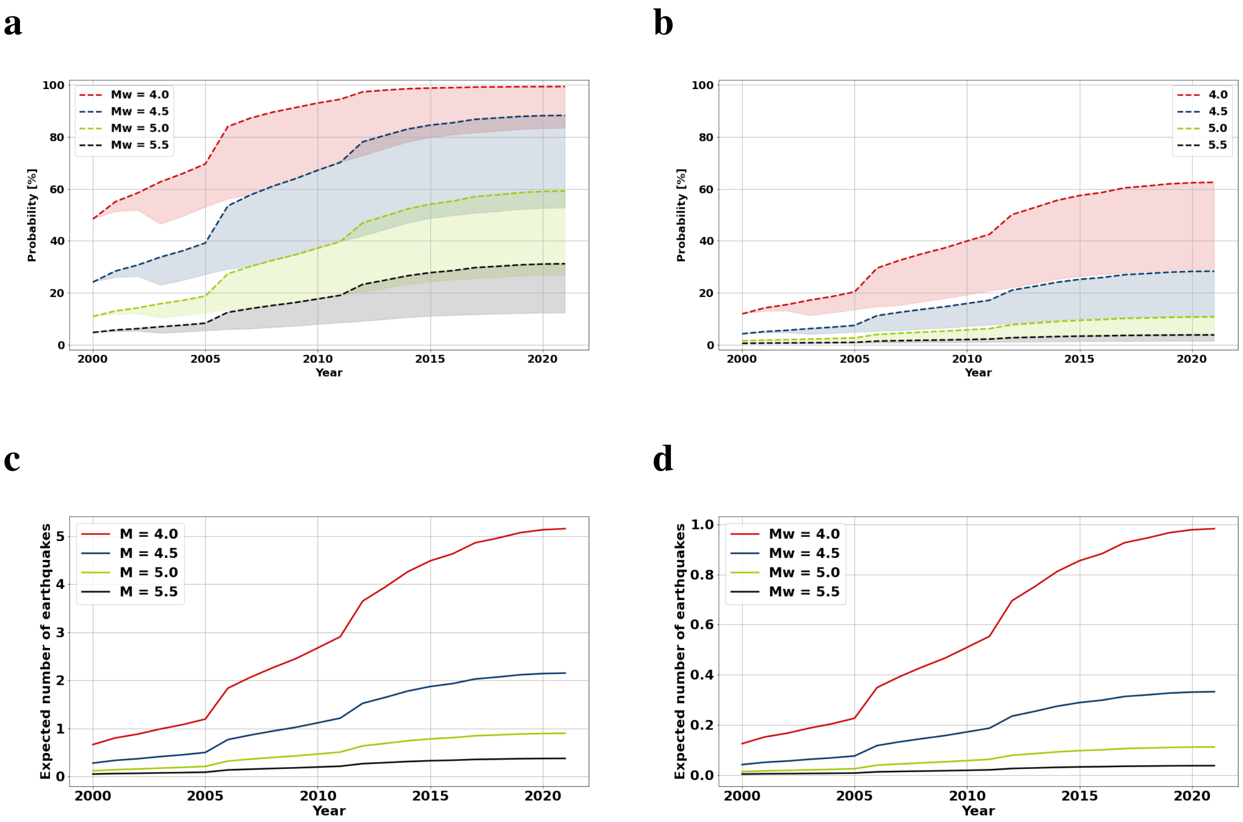

Supplementary Fig. 3: **Comparison of WCEPs using GR and LB statistics.** **a** LB-statistics **b** GR-statistics, **c** and **d** Expected number of earthquakes using probabilities from **a** and **b**. Triggering probabilities and the number of expected earthquakes is significantly larger for the LB model. Still, probabilities to trigger large-magnitude events are significant.

Is the lower-bound overfitting the data

Fitting the lower bound model to the Groningen FM-distribution provides a better fit than the GR-model. However, it uses one additional free parameter and is therefore expected to be better.

A similar question was investigated by Muntendam-Bos and Grobbe, 2022 who performed

a statistical analysis of the event-size distribution of the Groningen extraction-induced seismicity catalogue and find the probability of larger magnitude events in the NW-region is statistically significantly larger than in the southern and eastern parts of the gas field. However, the authors show how notoriously difficult it is to constrain a magnitude bound or taper from observed seismicity alone. We do not rely on the statistics of observed seismicity alone but include information about reservoir and rupture geometries into the analysis and find that an upper magnitude bound likely exists.

We used the Akaike Information Criterion (AIC) (Akaike, 1974) to assess if the GR-model or the LB-model is the preferred model. The AIC penalizes additional parameters in a considered model to prevent overfitting according to

$$AIC = -2log(L) + 2k \tag{1}$$

where log(L) is the loglikelihood of the model and k is its number of free parameters (k_{GR}=2,k_{LB}=3). Despite an additional parameter the LB-model is characterized by a lower AIC (lower information loss) in the magnitude range $M_{min} \geq 1.5$ and thus the preferred model (see Fig. S4).

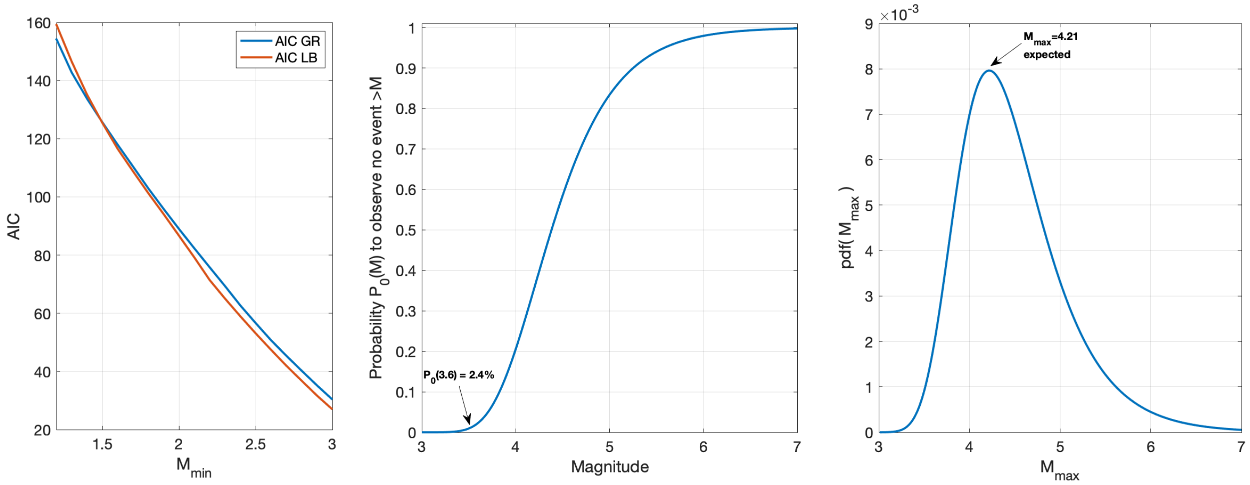

Supplementary Fig. 4: **Left:** Akaike Information Criterion (AIC) computed in magnitude ranges $M_{min} \leq M \leq 3.6$. Likelihood values for the GR- and LB-model fits are computed according to the Poisson assumption. Despite an additional parameter the LB-model is characterized by a lower AIC (lower information loss) in the magnitude range $M_{min} \geq 1.5$ and thus the preferred model. **Middle:** Probability to observe no event larger than a given magnitude M. The probability to observe no event larger than M=3.6 (the observed maximum magnitude at Groningen) is 2.3%. Probabilities are computed according to the classical GR-statistic fit and the Poisson assumption. **Right:** pdf of the maximum magnitude computed according to the classical GR-statistic and the Poisson assumption. The statistically expected maximum magnitude is given by M=4.21.

Comparison of different magnitude estimates

In the paper, we analyze the spatial distribution of the maximum observed magnitude (Mmax observed, Figure 1d), the maximum expected magnitude estimated from the reservoir thickness (Figure 1b) and the Mmax estimate from the fitting of the lower-bound equation (M_Y, Figure 3c). Figure S5 shows scatter plots of these three quantities for further analysis. The left plot shows the observed Mmax vs Mmax estimated from the thickness (for two different stress drops of 1 and 10MPa), the middle subplot the observed Mmax vs M_Y obtained from fitting the LB-equation and the right subplot Mmax from the thickness vs M_Y from fitting the LB-equation. Subplot a) shows a clear correlation between the observed magnitudes and reservoir thickness, which supports our hypothesis of reservoir limit ruptures. Subplots b) and c) show a positive correlation between the respective quantities, however, maximum magnitudes obtained from the lower-bound fit are significantly larger than observed and expected from the thickness. Nevertheless, this is a reasonable result as the LB provides an upperbound for the magnitude. An event of such magnitude might not have occurred yet.

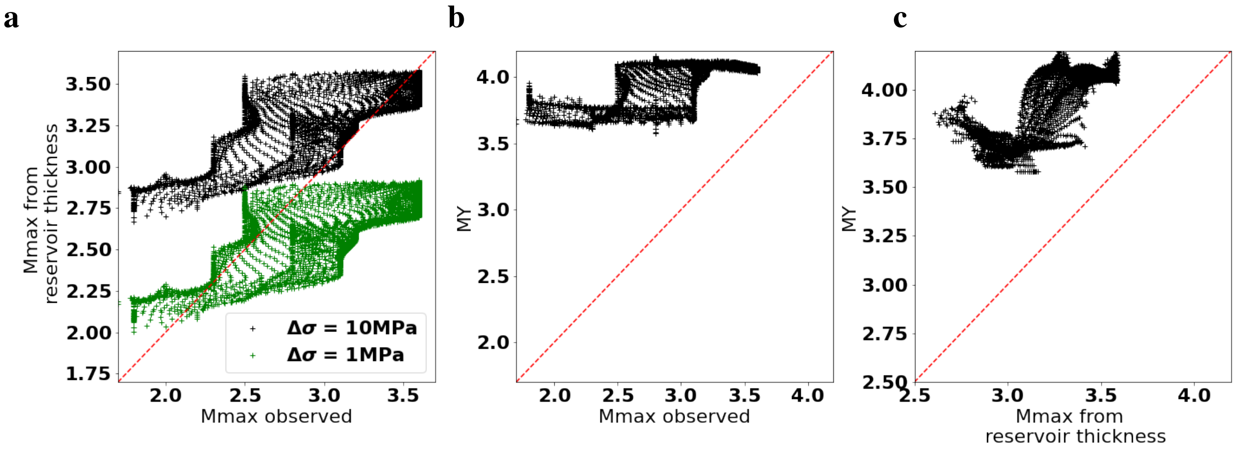

Supplementary Fig. 5: Scatter plots of different M_{Max} values. **a)** M_{Max} observed vs M_{Max} estimated from reservoir thickness, **b)** M_{Max} observed vs M_Y from the LB-fitting and **c)** M_{Max} from reservoir thickness vs M_Y. All three quantities show a positive correlation, M_Y show larger values, as they are upperbound limits.
