# Supplementary material for: Production-induced seismicity indicates a low risk of strong earthquakes in the Groningen gas field
Source: Nat Commun. 2024 Jan 6;15:329. doi: 10.1038/s41467-023-44485-4 (PMC10771524; doi:10.1038/s41467-023-44485-4)
Supplement: Supplementary file 3 — Supplementary Code [file 41467_2023_44485_MOESM3_ESM.zip › PDF_prints_scripts/Groningen_final_script_functions_publishing.pdf]

In [ ]:
```
import numpy as np
import matplotlib.pyplot as plt
from scipy.optimize import curve_fit



def eq_to_freq_mag_dist(eq_mag,lims = [.1,3.6]):
    mags = np.arange(lims[0],lims[1]+.1,.1)
    anz = np.zeros(np.size(mags))
    for idx,val in enumerate(mags):
        anz[idx] = np.sum(eq_mag >= val)
    return [mags,anz]

def eq_to_freq_mag_dist_1(eq_mag,lims = [.1,3.6]):
    mags = np.arange(lims[0],lims[1]+.1,.1)
    anz = np.zeros(np.size(mags))
    for idx,val in enumerate(mags):
        anz[idx] = np.sum(eq_mag >= val)

    i = 0
    while i < len(mags)-1:

        if anz[i] == anz[i+1]:
            mags = np.delete(mags,i)
            anz = np.delete(anz,i)
        else:
            i = i+1;

    return [mags,anz]

def maximum_likelihood_b(M):
    # from Wiemer and Wyss
    Mmin = np.min(M)-.05
    Mmean = np.mean(M)
    n = len(M)
    b = np.log10(np.exp(1))/(Mmean - Mmin)
    db = 2.3*b**2 * np.sqrt(sum((M-Mmean)**2)/(n*(n-1)))
    return b,db

def fit_GR(mags,anz,lims=[1.,3.]):
    anz = anz[mags>=lims[0]]
    mags = mags[mags>=lims[0]]
    anz = anz[mags<=lims[1]]
    mags = mags[mags<=lims[1]]
    [b,a] = np.polyfit(mags,np.log10(anz), 1)
    return [a,-b]

def LB_latest(M,a,b,c):
    #return a-b*M+2*np.log10(abs(1-10**((M-c)/2)))
    return a-b*M+2*np.log10(abs(1-10**((M-c)/2)))
def LB_exact(M,a,b,c):
    return a-b*M+np.log10(abs(1 -(2*b)/(2*b-1)*10**((M-c)/2)  + 1/(2*b-1)
*10**(b*(M-c)) ))


def GR_curvefit(M,a,b):
    return a-b*M
```

```python
def fit_GR_curvefit(mags,anz,lims=[1.,3.]):
    anz = anz[mags>=lims[0]]
    mags = mags[mags>=lims[0]]
    anz = anz[mags<=lims[1]]
    mags = mags[mags<=lims[1]]
    [c,coff] = curve_fit(GR_curvefit,mags,np.log10(anz))
    return [c,coff]


def fit_LB(mags,anz,lims=[1.,3.6],p_opt= [3.8,.7,4.]):
    anz = anz[mags>=lims[0]]
    mags = mags[mags>=lims[0]]
    anz = anz[mags<=lims[1]]
    mags = mags[mags<=lims[1]]
    [c,coff] =curve_fit(LB_latest,mags,np.log10(anz),p0 = p_opt)
    return [c,coff]

def fit_LB_exact(mags,anz,lims=[1.,3.6],p_opt= [3.8,.7,4.]):
    anz = anz[mags>=lims[0]]
    mags = mags[mags>=lims[0]]
    anz = anz[mags<=lims[1]]
    mags = mags[mags<=lims[1]]
    [c,coff] =curve_fit(LB_exact,mags,np.log10(anz),p0 = p_opt)
    return [c,coff]



def points_in_ellipse(x,phi,v1,v2,points):
    # Code inspired by https://math.stackexchange.com/questions/76457/che
ck-if-a-point-is-within-an-ellipse
    phi = np.deg2rad(phi+90)
    R = np.array([[np.cos(phi),np.sin(phi)],[-np.sin(phi),np.cos(phi)]]);
    r1 = np.transpose(np.array([1,0]))@R;
    r2 = np.transpose(np.array([0,1]))@R;
    Lambda = np.array([[1/(v1),0],[0,1/(v2)]])
    E = np.array([r2,r1])
    W = Lambda@np.transpose(E)
    is_inside = np.zeros(len(points))
    for i,p in enumerate(points):
        p_c = p - x
        n = W@p_c
        if np.linalg.norm(n) < 1:
            is_inside[i] = 1
    return is_inside
def plot_ellipse(x,phi,v1,v2,color):
    phi = (np.deg2rad(phi))
    theta_grid = np.linspace(0,2*np.pi);
    x_r  = v1*np.cos( theta_grid );
    y_r  = v2*np.sin( theta_grid );
    R = np.array([[np.cos(phi),np.sin(phi)],[-np.sin(phi),np.cos(phi)]]);
    r_ellipse = np.transpose(np.array([x_r,y_r]))@R;
    plt.plot(r_ellipse[:,0] + x[0],r_ellipse[:,1]+x[1],color)
    return [r_ellipse]
```
