# Supplementary material for: Production-induced seismicity indicates a low risk of strong earthquakes in the Groningen gas field
Source: Nat Commun. 2024 Jan 6;15:329. doi: 10.1038/s41467-023-44485-4 (PMC10771524; doi:10.1038/s41467-023-44485-4)
Supplement: Supplementary file 3 — Supplementary Code [file 41467_2023_44485_MOESM3_ESM.zip › PDF_prints_scripts/Groningen_final_script_publishing.pdf]

In [1]:
```
import pandas as pd
import numpy as np
import matplotlib.pyplot as plt
import Groningen as f1
from datetime import datetime
import warnings
warnings.filterwarnings('ignore')

fu_gruen = (153/255,204/255,0)
fu_blau = (0/255,51/255,102/255)
fu_rot = (204/255,0,0)
plt.rcParams.update({'font.size': 22})
plt.rcParams.update({'font.size': 22,'font.weight':'bold'})
```

Load Earthquake Catalogue and Field Outline

In [2]:
```
## Load Earthquake Catalogue
from datetime import datetime
data = pd.read_excel('./KNMI eventcatalogue 10 feb 2022.xlsx')
eq_east = data['Easting']
eq_north = data['Northing']
eq_year = data['Datum'].dt.year
eq_lin_time = data['Datum'].dt.year + data['Datum'].dt.month / 12 + data
['Datum'].dt.day / 365
eq_magnitude = data['Magnitude']

## Load Field Outline
f_outline = pd.read_csv('./field_outline.txt',delimiter='\t')
field_east = f_outline['X'][1:]
field_north = f_outline['Y'][1:]
```

Load Production Data

In [3]:
```
## Load Production Data
strange_wells = np.array([37,306,312,314,315,319,329,337,338,339,340,341,
342,343,344,345,346,347,348,349])
# Those wells show strange Pressure Curves
data = pd.read_csv('./Avg_OS1.csv')
locs = pd.read_csv('./station_code_and_loc.csv')

n_well = len(np.unique(data[' Well']))
n_times = len(np.unique(data['Date']))
wells = np.unique(data[' Well'])
wells = np.delete(wells,strange_wells)
d = {'stat_code': wells, 'east': np.ones(len(wells))*np.nan,'north': np.o
nes(len(wells))*np.nan}
df = pd.DataFrame(data=d)
east = np.array(locs['east'])
east = np.delete(east,strange_wells)
df['east'] = east
north = np.array(locs['north'])
north = np.delete(north,strange_wells)
df['north'] = north

## Build Production and Pressure Matrix
dates = np.unique(data['Date'])
prod_matrix = np.zeros([n_times,n_well])
prod_matrix[:] = np.nan
pressure_matrix = np.zeros([n_times,n_well])
pressure_matrix[:] = np.nan
count = 0;
for i_wells in range(n_well):
    for times in range(n_times):
        prod_matrix[times,i_wells] = data['Monthly Gas Production (M
3)'][count]
        pressure_matrix[times,i_wells] = data['Datum Pressure (BAR)'][cou
nt]
        count +=1
pressure_matrix = np.delete(pressure_matrix,strange_wells,axis=1)
prod_matrix = np.delete(prod_matrix,strange_wells,axis=1)
```

Annual Earthquake Rates and Production Rates

In [4]:
```
t = np.arange(1991,2022)
num = np.zeros(len(t))
M_max_obs = np.zeros(len(t))
for idx,i in enumerate(t):
    eq_dt = eq_magnitude[eq_year == i]
    num[idx] = np.sum(eq_dt >= 1.2)
    M_max_obs[idx] = np.max(eq_dt)

fig, ax1 = plt.subplots(figsize=(15,10) )
ax1.set_xlabel('Year',fontweight = 'bold')
ax1.bar(t, num,color = fu_blau)
ax1.tick_params(axis ='y', labelcolor = fu_blau)
ax1.set_ylabel('Annual earthquake rate (N>M$_C\,=\,$1.2)', color = fu_bla
u ,fontweight = 'bold')

ax2 = ax1.twinx()
ax2.plot(t, M_max_obs, color = fu_rot,lw=3)
ax2.tick_params(axis ='y', labelcolor = fu_rot)
ax2.set_ylabel('Maximum annual observed magnitude M$_L$', color = fu_rot,
fontweight = 'bold')
ax2.set_xlabel('Year',fontweight = 'bold')
```

Out[4]: Text(0.5, 0, 'Year')

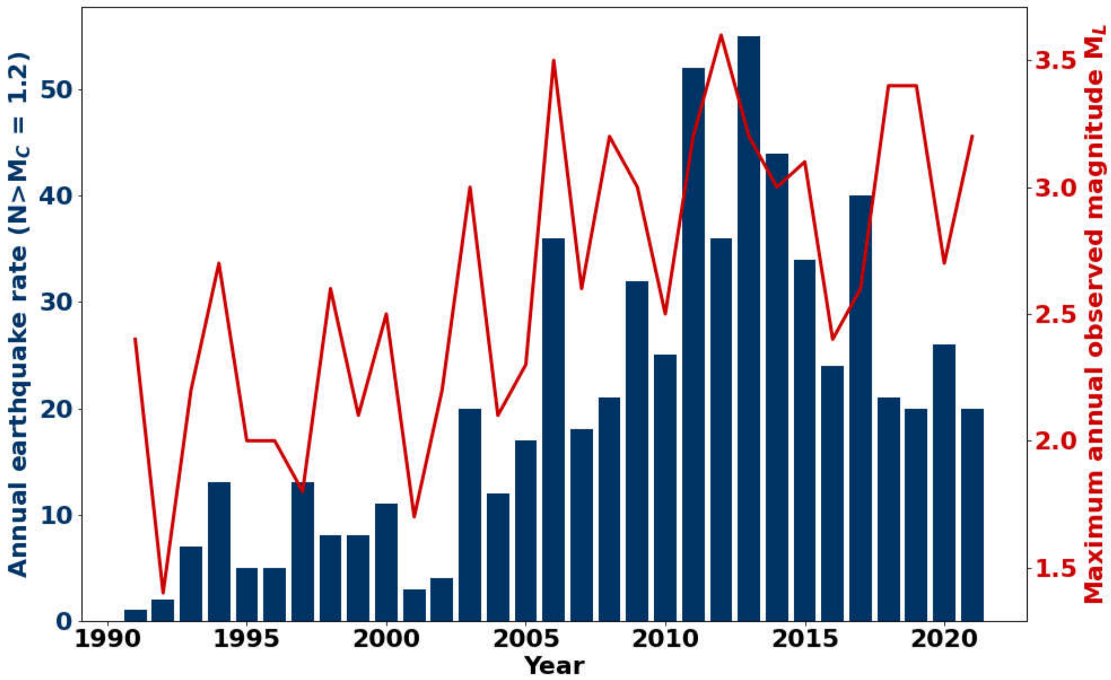

Annual Production Rates and Pressure Depletion

In [5]:
```
# Annual Production and Pressure
fig, ax1 = plt.subplots(figsize=(15,10) )
n_years = 2022-1957
annual_prod = np.zeros(n_years)
annual_pressure = np.zeros(n_years)
years = np.arange(1957,2022,1)
for times in range(n_years):
    annual_prod[times] = np.sum(prod_matrix[11 + times*12:23 + times*1
2,:])
    annual_pressure[times] = np.mean(pressure_matrix[11 + times*12:23 + t
imes*12,:])
ax1.bar(years,annual_prod/10**9,color = fu_blau)
ax1.set_xlim(1965,2022)
ax1.tick_params(axis ='y', labelcolor = fu_blau)
ax1.set_ylabel('Annual gas production [bcm]', color = fu_blau, fontweight
= 'bold')
ax2 = ax1.twinx()

pressure_derivative = -(annual_pressure[1:] - annual_pressure[0:-1])/10
ax2.plot(years[1:],pressure_derivative[:],color = fu_rot,lw=2.5)
ax2.tick_params(axis ='y', labelcolor = fu_rot)
ax2.set_ylabel('Annual Pressure Drop [MPa]', color = fu_rot, fontweight =
'bold')
ax1.set_xlabel('Year', fontweight = 'bold')
```

Out[5]: `Text(0.5, 0, 'Year')`

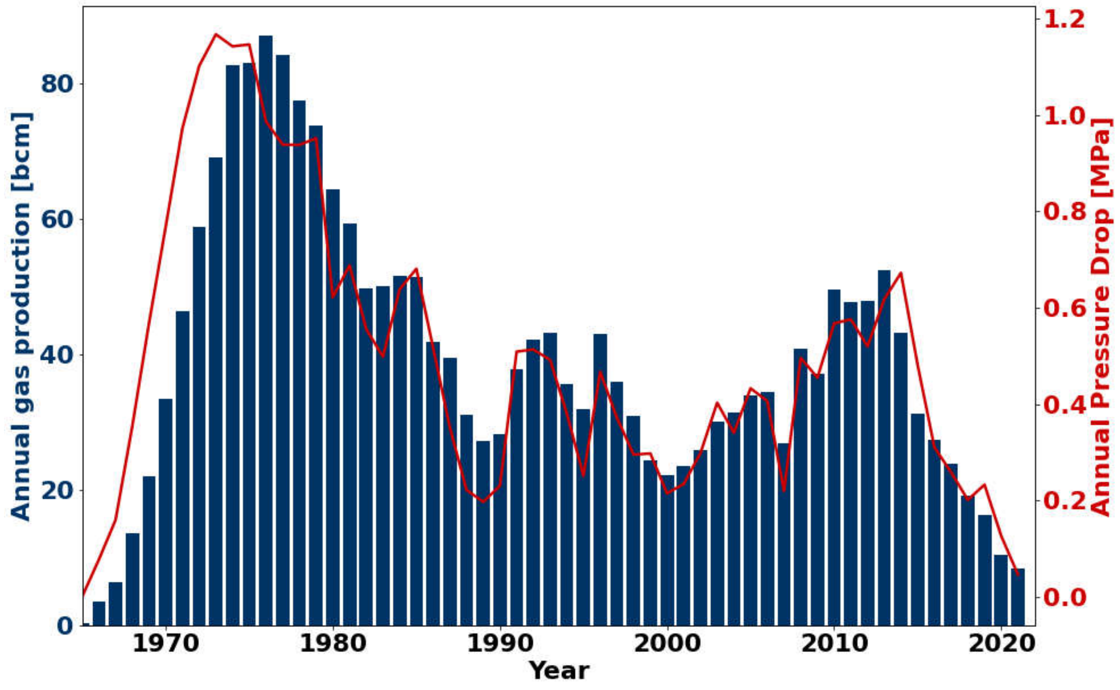

LB-Fit total time

In [6]:

```
MC = 1.2
mags = eq_magnitude
[mag,anz] = f1.eq_to_freq_mag_dist_1(mags,lims = [-.8,max(mags)])
b = f1.maximum_likelihood_b(mags[mags>MC])
[t,t_err_1] = f1.fit_GR_curvefit(mag,anz,lims=[MC,np.min([np.max(mags),3])])
xmag = np.arange(0,3.9,.1)
y_GR = f1.GR_curvefit(xmag,t[0],t[1])
[t_LB,err_LB_approx] = f1.fit_LB(mag,anz,lims=[MC,np.max(mags)],p_opt = [4,.9,max(mags)+.2])
y_LB = f1.LB_latest(xmag,t_LB[0],t_LB[1],t_LB[2])


[t_LB_exact,err_LB_exact] = f1.fit_LB_exact(mag,anz,lims=[MC,np.max(mags)],p_opt = [4,.9,max(mags)+.2])
y_LB_exact = f1.LB_exact(xmag,t_LB_exact[0],t_LB_exact[1],t_LB_exact[2])
plt.rcParams.update({'font.size': 18})
plt.figure(figsize=(15,8))
plt.plot(mag,np.log10(anz),'+',c=fu_blau,ms=10,mew=2,label='Data')
plt.plot(xmag,y_GR,'-',c=fu_rot,lw=2,label='GR-Fit')
plt.plot(xmag,y_LB_exact,'-',c=fu_blau,lw=2,label='Lower-Bound-Fit - Exact Equation')
plt.plot(xmag,y_LB,'--',c=fu_gruen,lw=2,label='Lower-Bound-Fit - Approximation')

plt.ylim([-0.05,4])
plt.xlim([-.25,4])
plt.ylabel('log10(N)',fontweight='bold')
plt.xlabel('Magnitude',fontweight='bold')
plt.legend()

plt.annotate('GR\n\na = ' + str(np.round(t[0],2)) + u"\u00B1" + str(round(np.sqrt(np.diag(t_err_1))[0],2)) + '\nb = ' + str(np.round(t[1],2))  + u"\u00B1" + str(round(np.sqrt(np.diag(t_err_1))[1],2)), xy=(1, 1),  xycoords='data',
            xytext=(0.0, 1.), verticalalignment='top',size=20,weight='bold')
plt.annotate('LB-exact\n\na = ' + str(np.round(t_LB_exact[0],2))+ u"\u00B1" + str(round(np.sqrt(np.diag(err_LB_exact))[0],2)) + '\nb = ' + str(np.round(t_LB_exact[1],2))+ u"\u00B1" + str(round(np.sqrt(np.diag(err_LB_exact))[1],2))+ '\nMy = ' + str(np.round(t_LB_exact[2],2))+ u"\u00B1" + str(round(np.sqrt(np.diag(err_LB_exact))[2],2)), xy=(1, 1),  xycoords='data',
            xytext=(1.15, 1.),verticalalignment='top',size=20,weight='bold')
plt.annotate('LB\n\na = ' + str(np.round(t_LB[0],2))  + u"\u00B1" + str(round(np.sqrt(np.diag(err_LB_approx))[0],2)) +'\nb = ' + str(np.round(t_LB[1],2)) + u"\u00B1" + str(round(np.sqrt(np.diag(err_LB_approx))[1],2)) + '\nMy = ' + str(np.round(t_LB[2],2)) + u"\u00B1" + str(round(np.sqrt(np.diag(err_LB_approx))[2],2)) , xy=(1, 1),  xycoords='data',
            xytext=(2.3, 1.),verticalalignment='top',size=20,weight='bold')
plt.show()
```

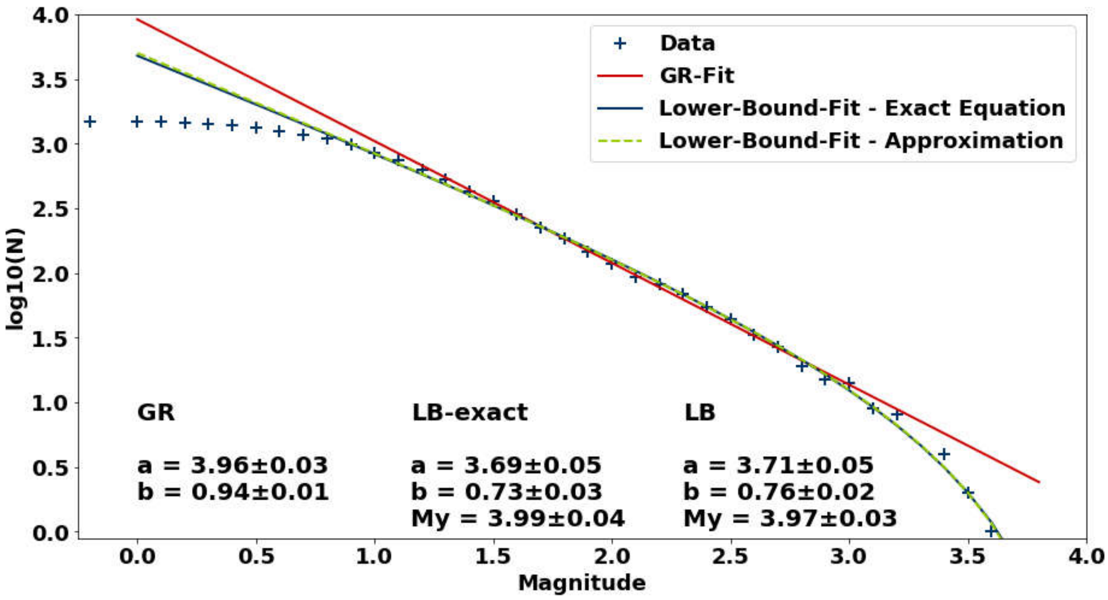

Temporal Seismogenic Index from pore-pressure

In [7]:
```
timespan = np.arange(2000,2021+1,1)

sigma_pp_total_t = np.zeros(len(timespan))
sigma_pp_total_t_GR = np.zeros(len(timespan))
sigma_pp_a_value_total_t = np.zeros(len(timespan))
sigma_pp_t0_1992 = np.zeros(len(timespan))
sigma_pp_a_value_t0_1992 = np.zeros(len(timespan))


dSigma_total_t = np.zeros(len(timespan))
#dSigma_total_t_GR = np.zeros(len(timespan))
dSigma_t0_1992 = np.zeros(len(timespan))
anz_time = np.zeros(len(timespan))

b_vals_GR = np.zeros(len(timespan))
b_vals_LB = np.zeros(len(timespan))
b_vals_GR_error = np.zeros(len(timespan))
b_vals_LB_error = np.zeros(len(timespan))

a_vals_GR = np.zeros(len(timespan))
a_vals_LB = np.zeros(len(timespan))
a_vals_GR_error = np.zeros(len(timespan))
a_vals_LB_error = np.zeros(len(timespan))


My = np.zeros(len(timespan))
My_error = np.zeros(len(timespan))


N_above_MC = np.zeros(len(timespan))

h = 250
A = 900*10**6
ns = 0.375
S = 5*10**(-10)
mu_f = 0.6
sin_phi = np.sin(np.arctan(mu_f))
dp = ((1-ns) - ns/sin_phi)
NC = 1.2
for idx,years in enumerate(timespan):

    mags = eq_magnitude[eq_year<=years]
    [mag,anz] = f1.eq_to_freq_mag_dist_1(mags,lims = [-.8,max(mags)])
    ## Gutenberg-Richter-Fit
    [t,t_err_1] = f1.fit_GR_curvefit(mag,anz,lims=[NC,3])
    a_vals_GR[idx] = t[0]
    a_vals_GR_error[idx] = t_err_1[0,0]
    b_vals_GR[idx] = t[1]
    b_vals_GR_error[idx] = t_err_1[1,1]
    ## Lower Bound Fit
    [t,t_err_1] = f1.fit_LB(mag,anz,lims=[NC,np.min([np.max(mags),3.6])])
    a_vals_LB[idx] = t[0]
    a_vals_LB_error[idx] = t_err_1[0,0]
    b_vals_LB[idx] = t[1]
    b_vals_LB_error[idx] = t_err_1[1,1]
    My[idx] = t[2]
    My_error[idx] = t_err_1[2,2]
    ## Get Pressure Drop
    dP_year = (annual_pressure[years-1957]-annual_pressure[0])/10
```

```
    dP_year_1992 = (annual_pressure[years-1957]-annual_pressure[1992-195
7])/10
    ## Seismogenic Index
    dSigma_total_t[idx] = np.log10(-A*h*dP_year*10**6) + np.log10(-dp*S)
    dSigma_t0_1992[idx] = np.log10(-A*h*dP_year_1992*10**6) + np.log10(-d
p*S)

    N = np.sum(mags>=NC)
    N_above_MC[idx] = N
    sigma_pp_total_t[idx] = np.log10(N) + NC*b_vals_LB[idx] - dSigma_tota
l_t[idx]
    sigma_pp_total_t_GR[idx] = np.log10(N) + NC*b_vals_GR[idx] - dSigma_t
otal_t[idx]
    sigma_pp_a_value_total_t[idx] = a_vals_LB[idx]-dSigma_total_t[idx]

    sigma_pp_t0_1992[idx] = np.log10(N) + NC*b_vals_LB[idx] - dSigma_t0_1
992[idx]
    sigma_pp_a_value_t0_1992[idx] = a_vals_LB[idx]-dSigma_t0_1992[idx]
```

Plot Temporal b-value

In [8]:
```
s_years = [2003,2019] # 100 and 600 Events above MC
b_uncerti = np.array([0.13,0.07])
my_uncerti = np.array([0.73,0.17])
line_upper = np.polyfit(s_years,.76 + b_uncerti,1)
yb1 = np.polyval(line_upper,[2000,2021])
line_lower = np.polyfit(s_years,.76 - b_uncerti,1)
yb2 = np.polyval(line_lower,[2000,2021])

line_upper = np.polyfit(s_years,3.97 + my_uncerti,1)
ym1 = np.polyval(line_upper,[2000,2021])
line_lower = np.polyfit(s_years,3.97 - my_uncerti,1)
ym2 = np.polyval(line_lower,[2000,2021])

## b-value - GR and LB over time
plt.figure(figsize=(15,8))
plt.errorbar(timespan,b_vals_GR,yerr = np.sqrt(b_vals_GR_error),color = f
u_blau,lw=2.5,label='GR-fit')
plt.errorbar(timespan,b_vals_LB,yerr = np.sqrt(b_vals_LB_error),color = f
u_rot,lw=2.5,label='LB-fit')
plt.fill(np.array([2000,2021,2021,2000,2000]),np.array([yb1[0],yb1[1],yb2
[1],yb2[0],yb1[0]]),color=fu_rot,alpha=.5)
plt.legend()
plt.xlabel('Year',fontweight = 'bold')
plt.ylabel('b-value',fontweight = 'bold')
plt.grid('minor')
```

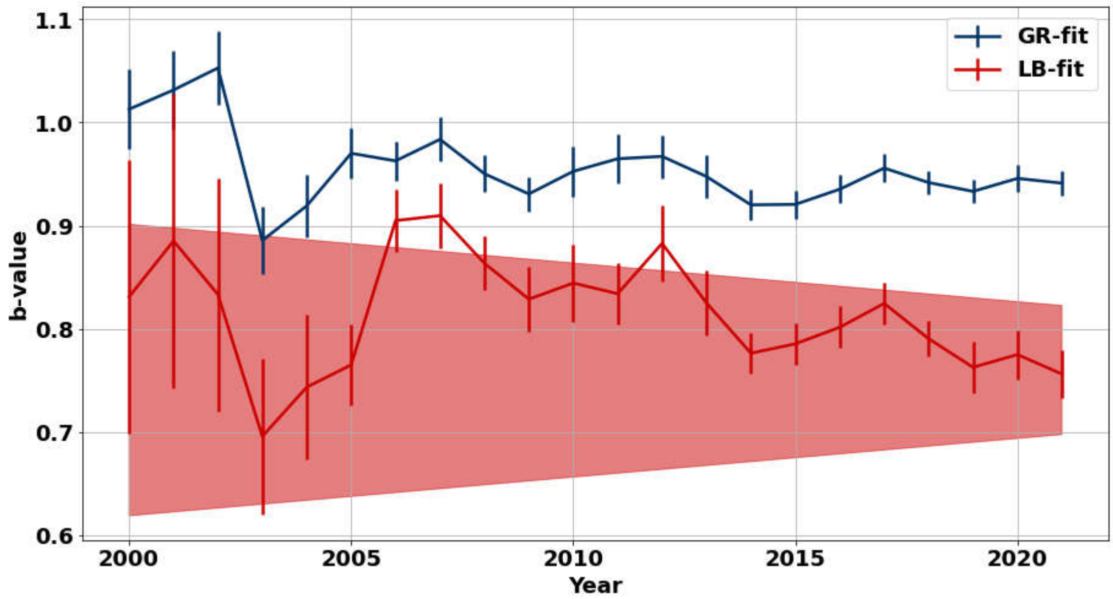

In [9]:
```
## a-value - GR and LB over time

fig, ax1 = plt.subplots(figsize=(15,8) )
ax1.errorbar(timespan,a_vals_GR,yerr = np.sqrt(a_vals_GR_error),color = f
u_blau,lw=2.5,label='GR-fit')
ax1.errorbar(timespan,a_vals_LB,yerr = np.sqrt(a_vals_LB_error),color = f
u_rot,lw=2.5,label='LB-fit')
plt.legend()
plt.xlabel('Year',fontweight = 'bold')
ax1.tick_params(axis ='y', labelcolor = fu_blau)
ax1.set_ylabel('a-value', color = fu_blau ,fontweight = 'bold')

ax2 = ax1.twinx()
ax2.plot(timespan,N_above_MC,color=fu_gruen,lw=2.5)
ax2.tick_params(axis ='y', labelcolor = fu_gruen)
ax2.set_ylabel('# events above MC=1.2', color = fu_gruen ,fontweight = 'b
old')
plt.grid('minor')
```

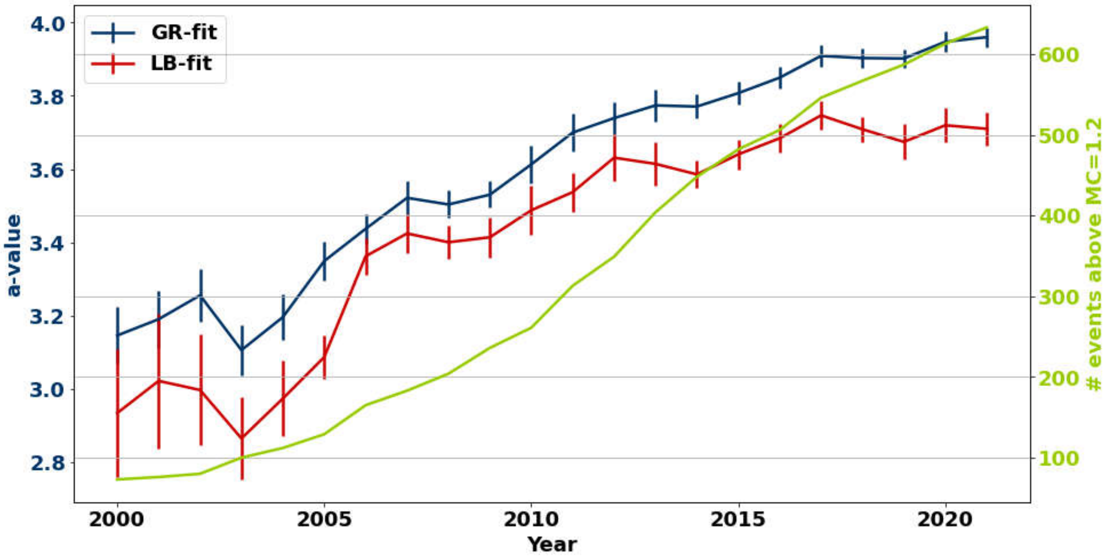

In [10]:
```
## My over time
plt.figure(figsize=(15,8))
plt.errorbar(timespan,My,yerr = np.sqrt(My_error),color = fu_blau,lw=2.5,
label='LB-fit')
plt.fill(np.array([2000,2021,2021,2000,2000]),np.array([ym1[0],ym1[1],ym2
[1],ym2[0],ym1[0]]),color=fu_blau,alpha=.5)
plt.xlabel('Year',fontweight = 'bold')
plt.ylabel('My',fontweight = 'bold')
plt.grid('minor')
```

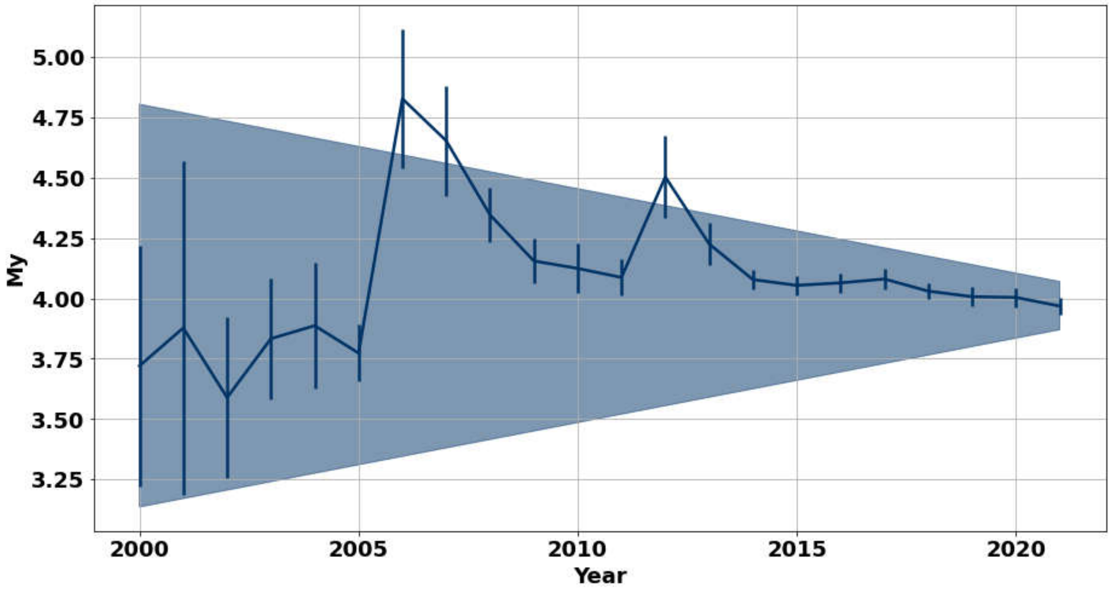

In [11]:
```
## Seismogenic Index over time
plt.figure(figsize=(15,8))
plt.errorbar(timespan,sigma_pp_total_t,yerr = np.sqrt(a_vals_LB_error),co
lor = fu_gruen,lw=2.5,label='$\Sigma_0$[EQ 10] T$_{1963}$')
plt.plot(timespan,sigma_pp_a_value_total_t,'--',color = fu_gruen,lw=2.5,l
abel='$\Sigma_0$[EQ 9] T$_{1963}$')
plt.errorbar(timespan,sigma_pp_t0_1992,yerr = np.sqrt(a_vals_LB_error),co
lor = fu_blau,lw=2.5,label='$\Sigma_0$[EQ 10] T$_{1963}$')
plt.plot(timespan,sigma_pp_a_value_t0_1992,'--',color = fu_blau,lw=2.5,la
bel='$\Sigma_0$[EQ 9] T$_{1992}$')
plt.xlabel('Year',fontweight = 'bold')
plt.ylabel('Seismogenic Index',fontweight = 'bold')
plt.legend()
plt.grid('minor')
```

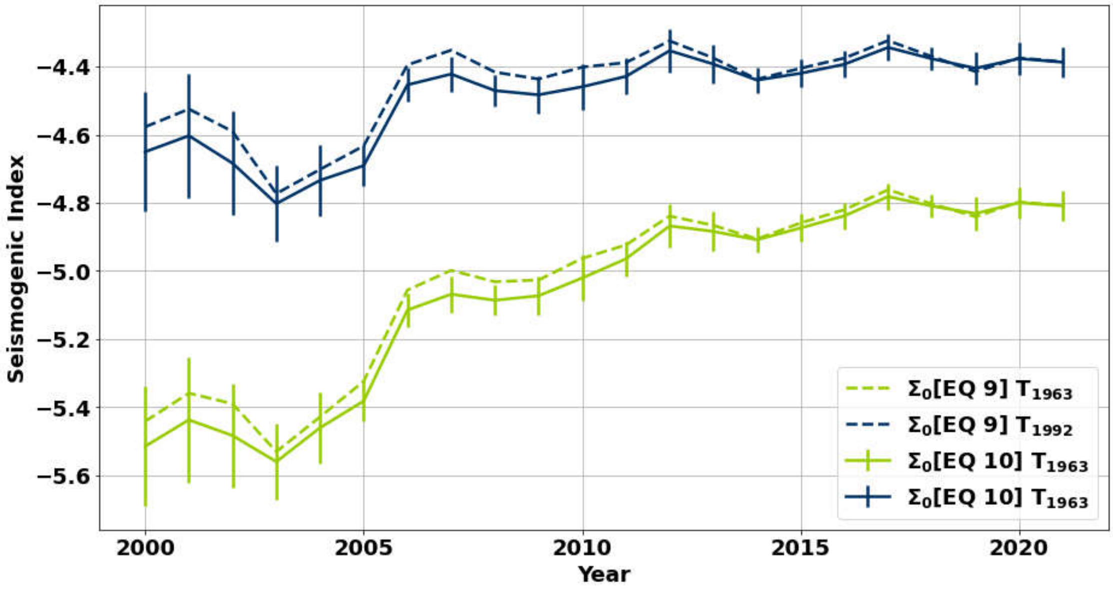

Triggering Probabilities

In [12]:

```
m_trigger = np.arange(4,6,.5)
colors = [fu_rot,fu_blau,fu_gruen,[0,0,0]]
### LB - WCEP
plt.figure(figsize=(15,8))
b_vals_LB[:] = .76
for jdx,M in enumerate(m_trigger):
    prob_pp = np.zeros(len(timespan))
    prob_pp_upper = np.zeros(len(timespan))
    prob_pp_lower = np.zeros(len(timespan))
    prob_pp_1992 = np.zeros(len(timespan))
    for idx,years in enumerate(timespan):
        prob_pp_1992[idx] = 1 - np.exp(-10**(dSigma_t0_1992[idx] +  np.ma
x(sigma_pp_t0_1992[0:idx+1]) - np.min(b_vals_LB[0:idx+1]) * M))
        prob_pp_upper[idx] = 1 - np.exp(-10**(dSigma_t0_1992[idx] +  np.m
ax(sigma_pp_t0_1992[0:idx+1]) - np.min(b_vals_LB[0:idx+1]) * M))
        prob_pp_lower[idx] = 1 - np.exp(-10**(dSigma_t0_1992[idx] +  np.m
in(sigma_pp_t0_1992[0:idx+1]) - np.max(b_vals_LB[0:idx+1]) * M))
        prob_pp[idx] = 1 - np.exp(-10**(dSigma_total_t[idx] +  np.max(sig
ma_pp_total_t[0:idx+1]) - np.min(b_vals_LB[0:idx+1]) * M))
    plt.plot(timespan,prob_pp_upper*100,'-',lw=2.5,c = colors[jdx],label=
('Mw = '+ str(M)))
plt.xlabel('Year',fontweight = 'bold')
plt.ylabel('Probability [%]',fontweight = 'bold')
plt.legend()
plt.grid('minor')
plt.ylim([-2,102])

## Number of expected Earthquakes - LB
plt.figure(figsize=(15,8))
for jdx,M in enumerate(m_trigger):
    recurrence = np.zeros(len(timespan))
    for idx,years in enumerate(timespan):
        prob_pp[idx] = 1 - np.exp(-10**(dSigma_t0_1992[idx] +  np.max(sig
ma_pp_t0_1992[0:idx+1]) - np.min(b_vals_LB[0:idx+1]) * M))
        recurrence[idx] = -np.log(1-prob_pp[idx])
    plt.plot(timespan,recurrence,lw=2.5,label=('M = '+ str(M)),c = colors
[jdx])
plt.xlabel('Year',fontweight = 'bold')
plt.ylabel('Expected number of earthquakes',fontweight = 'bold')
plt.legend()
plt.grid('minor')
```

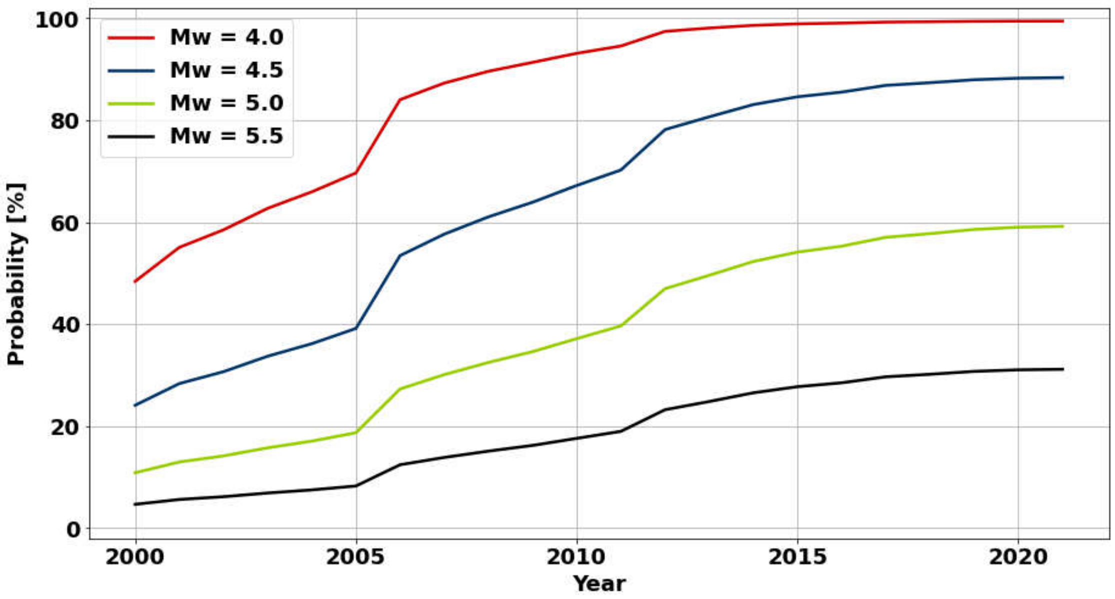

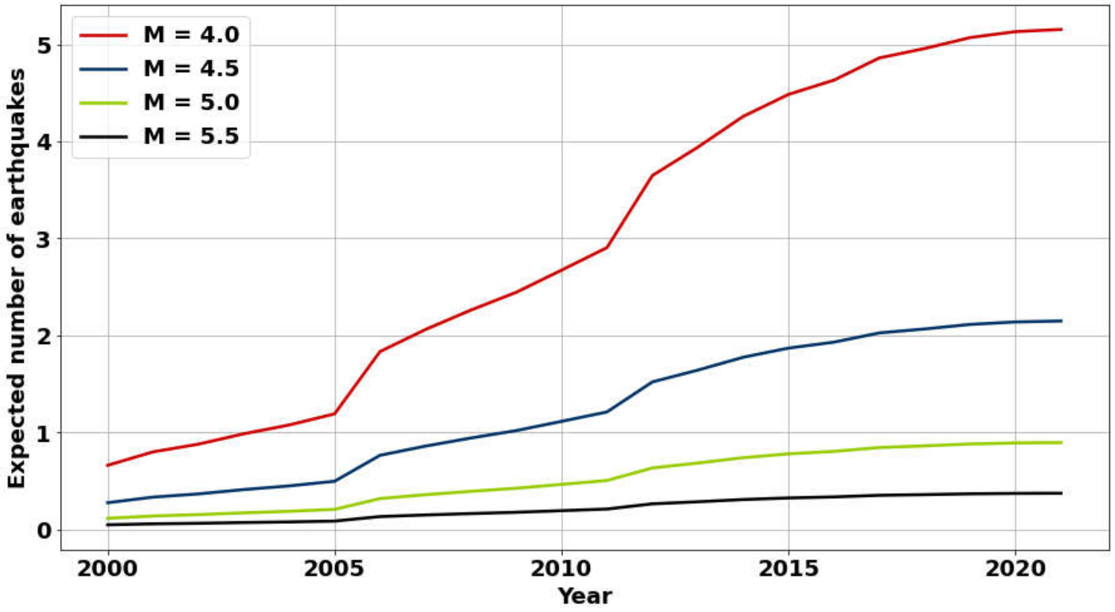

Van der Elst - Mmax

In [13]:
```
plt.figure(figsize=(15,8))
van_der_elst_M_LB = np.zeros(len(timespan))
van_der_elst_M_GR = np.zeros(len(timespan))
b_vals_GR[:] = .935
b_vals_LB[:] = .76

for idx,years in enumerate(timespan):
    van_der_elst_M_LB[idx] = 1/b_vals_LB[idx] * (sigma_pp_total_t[idx] + 
dSigma_total_t[idx])
    van_der_elst_M_GR[idx] = 1/b_vals_GR[idx] * (sigma_pp_total_t_GR[idx] 
+ dSigma_total_t[idx])
plt.plot(timespan,van_der_elst_M_GR,lw=2.5,c = colors[0],label='GR-b-valu
e')
plt.plot(timespan,van_der_elst_M_LB,lw=2.5,c = colors[1],label='LB-b-valu
e')
plt.xlabel('Year',fontweight = 'bold')
plt.ylabel('Expected maximum magnitude',fontweight = 'bold')
plt.legend()
plt.grid('minor')
```

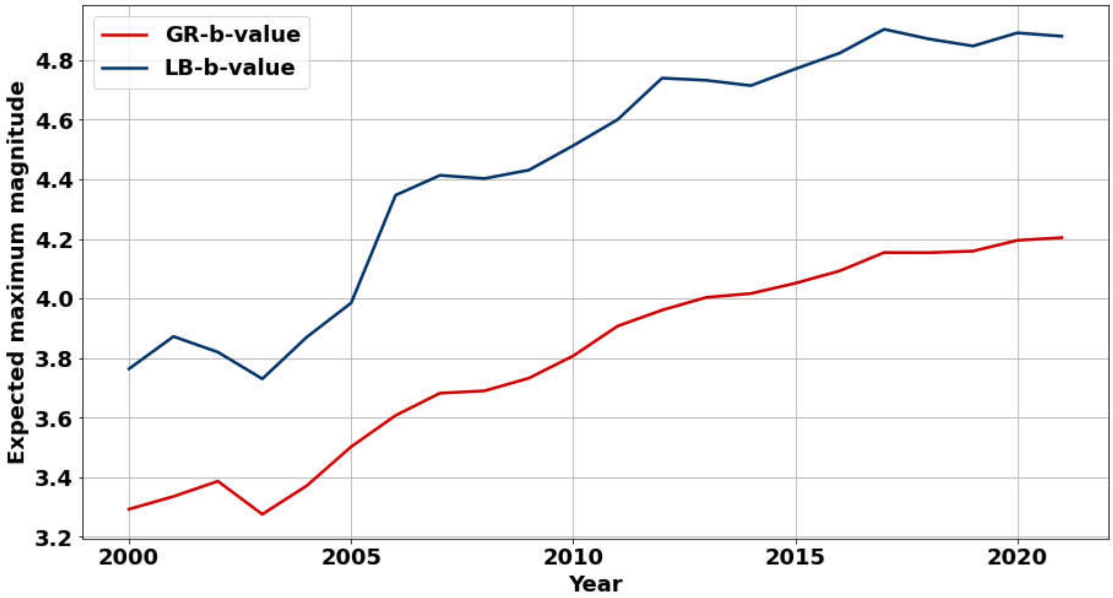

Spatial Parameter mapping

Reservoir Thickness

In [14]:
```
## Load reservoir Top and bottom from reflection seismics
top = np.loadtxt('./RO_T.txt')
bottom = np.loadtxt('./DC_T.txt')
outline = np.loadtxt('./Groningen_Field_outline.txt')
```

In [15]:
```
min_x = 230000#min(top[:,2])
min_y = 560000#min(top[:,3])
max_x = 270000#max(top[:,2])
max_y = 615000#max(top[:,3])
dx = 500
easti = np.arange(min_x,max_x,dx)
northi = np.arange(min_y,max_y,dx)
thick_spatial = np.zeros([len(easti)+1,len(northi)+1])
thick_spatial[:] = np.nan
top_spatial = np.zeros([len(easti)+1,len(northi)+1])
top_spatial[:] = np.nan
for i in range(np.shape(top)[0]):
    thick_spatial[int((top[i,2]-min_x)/dx),int((top[i,3]-min_y)/dx)] = -t
op[i,4] + bottom[i,4]
    top_spatial[int((top[i,2]-min_x)/dx),int((top[i,3]-min_y)/dx)] = -top
[i,4]
#my_spatial_theoretical = np.log10(thick_spatial**2) + np.log10(stress_dr
op)/1.5 - 6.07

np.shape(thick_spatial)
```

Out[15]:
```
(81, 111)
```

In [16]:
```
stress_drop = 10**7
easti = np.arange(min_x,max_x,dx)
my_spatial_theoretical = np.log10(thick_spatial**2) + np.log10(stress_dro
p)/1.5 - 6.07
plt.figure(figsize=(10,9))
plt.imshow(np.flipud(np.transpose(my_spatial_theoretical)),extent = [min_
x-dx/2,max_x-dx/2,min_y-dx/2,max_y-dx/2])
plt.xlim([232500,267500])
plt.ylim([570000,612500])
plt.colorbar()
plt.plot(outline[:,0],outline[:,1],'r-')
plt.show()
```

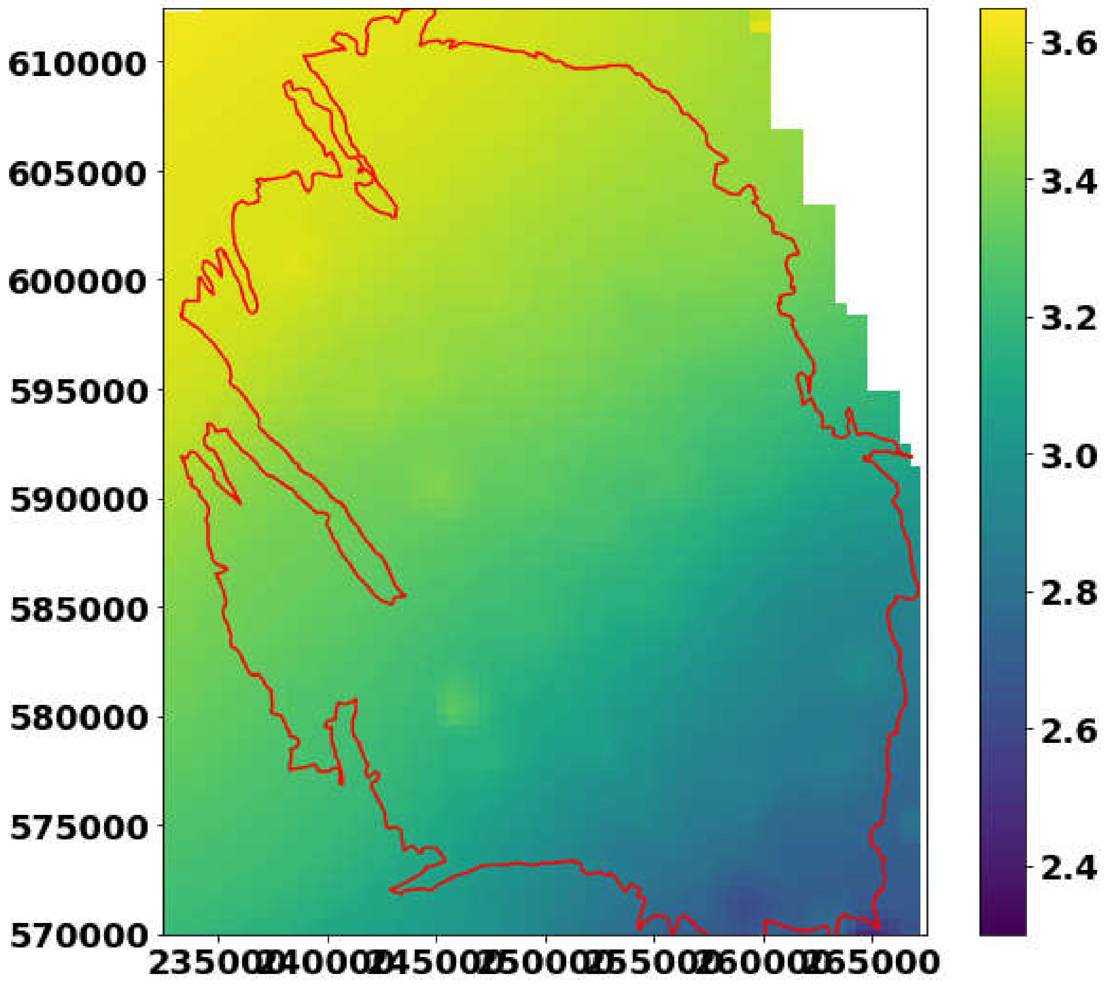

In [17]:
```
def points_in_ellipse(x,phi,v1,v2,points):
    # Code inspired by https://math.stackexchange.com/questions/76457/che
ck-if-a-point-is-within-an-ellipse
    phi = np.deg2rad(phi+90)
    R = np.array([[np.cos(phi),np.sin(phi)],[-np.sin(phi),np.cos(phi)]]);
    r1 = np.transpose(np.array([1,0]))@R;
    r2 = np.transpose(np.array([0,1]))@R;
    Lambda = np.array([[1/(v1),0],[0,1/(v2)]])
    E = np.array([r2,r1])
    W = Lambda@np.transpose(E)
    is_inside = np.zeros(len(points))
    for i,p in enumerate(points):
        p_c = p - x
        n = W@p_c
        if np.linalg.norm(n) < 1:
            is_inside[i] = 1
    return is_inside
xlim = [230000,270000]
ylim = [560000,615000]
dx = 500;
dy = 500;
x = np.arange(xlim[0],xlim[1],dx)
y = np.arange(ylim[0],ylim[1],dy)
max_obs_spatial = np.zeros([len(x),len(y)])
max_obs_spatial[:] = np.nan
east = eq_east
north = eq_north
eq_pos = np.transpose(np.array([east,north]))
mags = eq_magnitude
n_eq = 500
n_count = 0
for idx_x,x_s in enumerate(x):
    for idx_y,y_s in enumerate(y):
        eq_subset = [0,0];
        eq_pos_t = np.array([[x_s,y_s],[x_s,y_s]])
        if sum(f1.points_in_ellipse([250500,590000],-65,22000*.8,16000*.
7,eq_pos_t)) < 1:
            continue
        eq_subset = points_in_ellipse([x_s,y_s],0,dx*3*5,dy*3*5,eq_pos)
        mags_sub = mags[eq_subset==1]
        if len(mags_sub)> 0:
            max_obs_spatial[idx_x,idx_y] = max(mags_sub)
        else:
            max_obs_spatial[idx_x,idx_y] = np.nan
sm_base = 5
circle = np.zeros([2*sm_base,2*sm_base])
for i in range(2*sm_base):
    for j in range(2*sm_base):
        if np.sqrt((i-sm_base)**2 + (j-sm_base)**2) <= sm_base:
            circle[i,j] = 1
idx = np.where(circle==1)
max_obs_spatial_smooth = np.zeros(np.shape(max_obs_spatial))
max_obs_spatial_smooth[:] = np.nan
for i in range(sm_base,np.shape(max_obs_spatial)[0]-sm_base):
    for j in range(sm_base,np.shape(max_obs_spatial)[1]-sm_base):
        max_obs_spatial_smooth[i,j] = np.nanmean(max_obs_spatial[idx
[0][:] + i-sm_base,idx[1][:] + j-sm_base])

plt.figure(figsize=(10,9))
```

```
plt.imshow(np.flipud(np.transpose(max_obs_spatial_smooth)),extent = [min_
x-dx/2,max_x-dx/2,min_y-dx/2,max_y-dx/2])
plt.plot(outline[:,0],outline[:,1],'r-')
plt.plot(eq_east,eq_north,'k+',alpha=.15)
plt.colorbar()
plt.show()
```

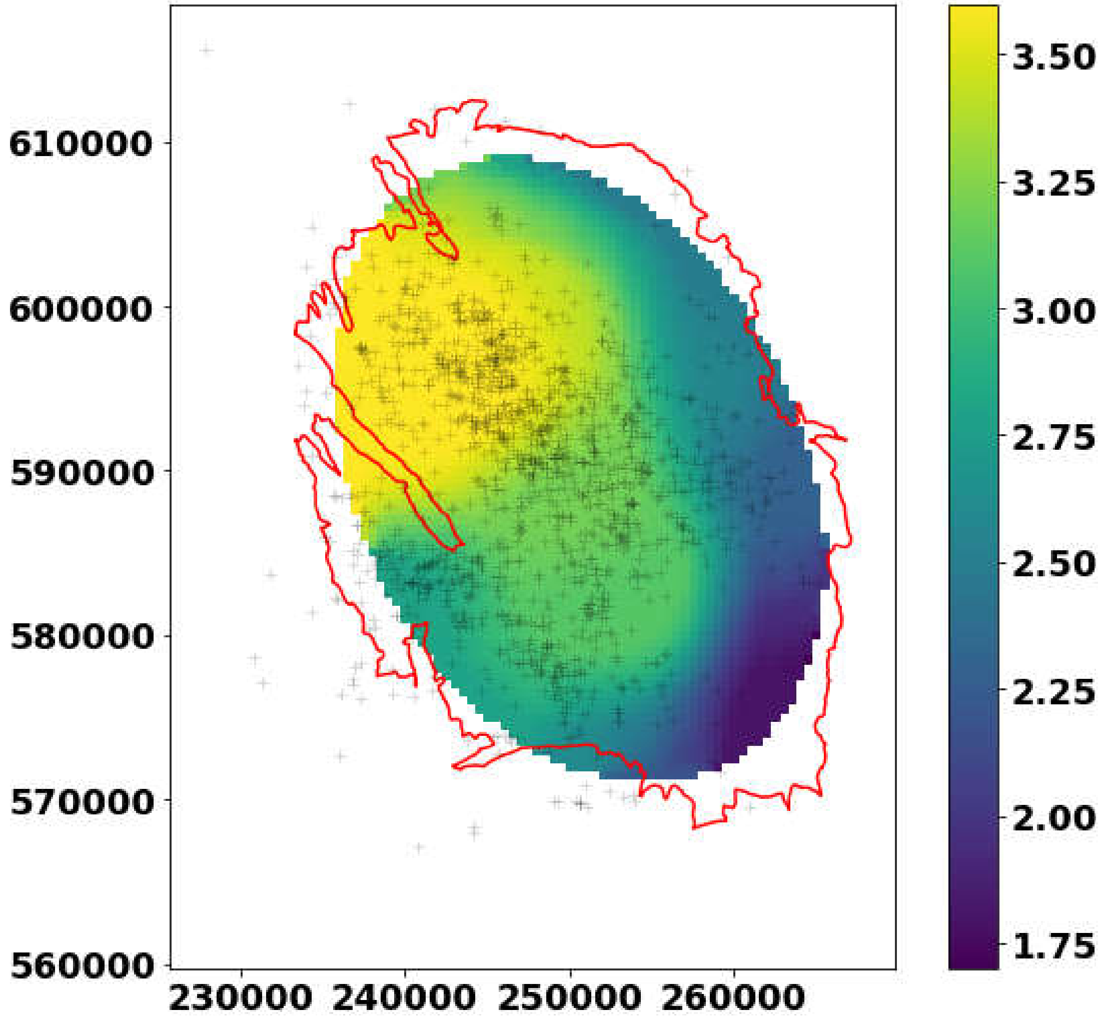

Estimate MMmax from reservoir thickness

In [18]:
```
my_spatial_theoretical = np.zeros([len(easti)+1,len(northi)+1])
stress_drop = 10**7 # 10 MPa
my_spatial_theoretical = np.log10(thick_spatial**2) + np.log10(stress_dro
p)/1.5 - 6.07
my_spatial_theoretical_lower = np.log10(thick_spatial**2) + np.log10(stre
ss_drop/10)/1.5 - 6.07
plt.figure(figsize=(10,9))
plt.imshow(np.flipud(np.transpose(my_spatial_theoretical)),extent = [min_
x-dx/2,max_x-dx/2,min_y-dx/2,max_y-dx/2])
plt.plot(field_east,field_north,'r-')
plt.xlim([232500,267500])
plt.ylim([570000,612500])
plt.colorbar()
plt.xticks([235000,245000,255000,265000])
plt.xlabel('Easting',fontweight = 'bold')
plt.ylabel('Northing',fontweight = 'bold')
#plt.savefig('../Paper/2023 - Groningen/Fig/Mfromthickness.png')
```

Out[18]:
```
Text(0, 0.5, 'Northing')
```

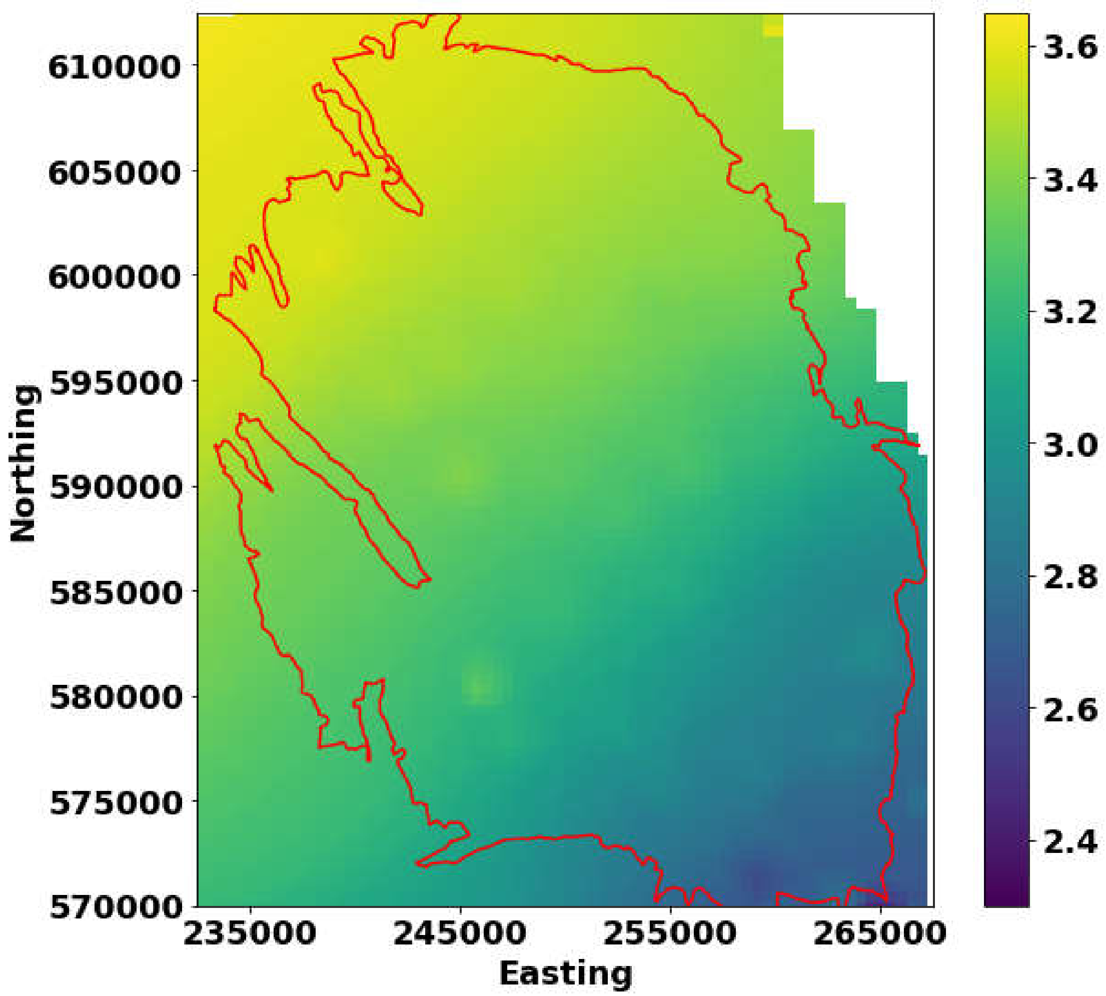

In [19]:
```
## Lateral Seismogenic Index - Compute a and b Values
def idx_of_nearest_earthquakes(pos,quakes,n_eq=200):
    r = np.sqrt((eq_east - pos[0])**2 + (eq_north - pos[1])**2)
    idx = np.argsort(r)
    #print(r)
    #print(r[idx])
    return idx[:n_eq]


def distance_from_center(x,phi,v1,v2,points):
    # Code inspired by https://math.stackexchange.com/questions/76457/che
ck-if-a-point-is-within-an-ellipse
    phi = np.deg2rad(phi+90)
    R = np.array([[np.cos(phi),np.sin(phi)],[-np.sin(phi),np.cos(phi)]]);
    r1 = np.transpose(np.array([1,0]))@R;
    r2 = np.transpose(np.array([0,1]))@R;
    Lambda = np.array([[1/(v1),0],[0,1/(v2)]])
    E = np.array([r2,r1])
    W = Lambda@np.transpose(E)
    is_inside = np.zeros(len(points))
    for i,p in enumerate(points):
        p_c = p - x
        n = W@p_c
        return np.linalg.norm(n)


east_lims = [230000,270000]
north_lims = [560000,615000]
disc = 250
global_sig = int(1)#; *disctre
offset = global_sig*6;
matrix = np.zeros([n_years,round((north_lims[1]-north_lims[0])/disc)+2*of
fset,round((east_lims[1]-east_lims[0])/disc)+2*offset])


eastcoord = np.arange(east_lims[0]-offset*disc,east_lims[1]+offset*disc,d
isc)#[east_lims[0]-offset*disc,east_lims[1]+offset*disc,north_lims[0]-off
set*disc
northcoord = np.arange(north_lims[0]-offset*disc,north_lims[1]+offset*dis
c,disc)

sigma_lateral = np.zeros([np.shape(matrix)[1],np.shape(matrix)[2]])
sigma_pp_lateral = np.zeros([np.shape(matrix)[1],np.shape(matrix)[2]])
d_sigma_lateral = np.zeros([np.shape(matrix)[1],np.shape(matrix)[2]])
stress_drop_lateral = np.zeros([np.shape(matrix)[1],np.shape(matrix)[2]])
M_max_lateral = np.zeros([np.shape(matrix)[1],np.shape(matrix)[2]])
a_lateral = np.zeros([np.shape(matrix)[1],np.shape(matrix)[2]])
a_lateral_GR = np.zeros([np.shape(matrix)[1],np.shape(matrix)[2]])
My_lateral = np.zeros([np.shape(matrix)[1],np.shape(matrix)[2]])
b_lateral = np.zeros([np.shape(matrix)[1],np.shape(matrix)[2]])
b_lateral_GR = np.zeros([np.shape(matrix)[1],np.shape(matrix)[2]])
dist_from_center = np.zeros([np.shape(matrix)[1],np.shape(matrix)[2]])

My_lateral_uncertainy = np.zeros([np.shape(matrix)[1],np.shape(matri
x)[2]])
My_lateral_uncertainy[:] = np.nan

a_lateral_uncertainy = np.zeros([np.shape(matrix)[1],np.shape(matri
x)[2]])
```

```
a_lateral_uncertainy[:] = np.nan

b_lateral_uncertainy = np.zeros([np.shape(matrix)[1],np.shape(matri
x)[2]])
b_lateral_uncertainy[:] = np.nan



stress_drop_lateral[:] = np.nan
dist_from_center[:] = np.nan
sigma_pp_lateral[:] = np.nan
M_max_lateral[:] = np.nan
a_lateral[:] = np.nan
b_lateral[:] = np.nan
a_lateral_GR[:] = np.nan
b_lateral_GR[:] = np.nan
d_sigma_lateral[:] = np.nan

My_lateral[:] = np.nan

R = 15000 # Radius in m
N_eq = 500
eq_pos = np.transpose(np.array([eq_east,eq_north])) # Earthquake Hypocent
ers

eq_pos = np.transpose(np.vstack((np.array(eq_east),np.array(eq_north))))

# Seismogenic Index Parameters Serge
A = 900*10**6#np.pi*R**2
thick = 300;
ns = 0.375
S = 5*10**(-10)
mu_f = 0.6
sin_phi = np.sin(np.arctan(mu_f))
dp = ((1-ns) - ns/sin_phi)
DP =   (annual_pressure[2020-1957]-annual_pressure[0])/10
MC = 1.2


for idx,e_pos in enumerate(eastcoord):
    for jdx,n_pos in enumerate(northcoord):
        eq_pos_t = np.array([[e_pos,n_pos],[e_pos,n_pos]])
        if sum(f1.points_in_ellipse([250500,590000],-65,22000*.8,16000*.
7,eq_pos_t)) < 1:
            continue
        r = np.sqrt((eq_pos[:,0] - e_pos)**2 + (eq_pos[:,1] - n_pos)**2)
        posi = r<=R
        dist_from_center[jdx,idx] = distance_from_center([250000,59000
0],-65,22000*.8,16000*.7,eq_pos_t)
        [freq,mag] = f1.eq_to_freq_mag_dist_1(eq_magnitude[posi],lims =
[np.min(eq_magnitude),np.max(eq_magnitude[posi])-.1])
        mag = mag[freq>=MC]
        freq = freq[freq>=MC]
        M_max_lateral[jdx,idx] = max(eq_magnitude[posi])
        mag = mag[freq<np.min([np.max(mag),np.max(eq_magnitude[posi])-.
1])]
        freq = freq[freq<np.min([np.max(mag),np.max(eq_magnitude[posi])-.
1])]
        [C,dummy] = f1.fit_LB(freq,mag,lims=[MC,np.max(eq_magnitude[pos
i])-.1])
```

```
        My_lateral[jdx,idx] = C[2]
        a_lateral[jdx,idx] = C[0]
        b_lateral[jdx,idx] = C[1]
        My_lateral_uncertainy[jdx,idx] = dummy[2,2]
        a_lateral_uncertainy[jdx,idx] = dummy[0,0]
        b_lateral_uncertainy[jdx,idx] = dummy[1,1]


        [c,coff] = f1.fit_GR_curvefit(freq,mag,lims=[MC,np.max(eq_magnitu
de[posi])-.1])
        a_lateral_GR[jdx,idx] = c[0]
        b_lateral_GR[jdx,idx] = c[1]

        tt = thick_spatial[np.argmin(abs(easti - e_pos)),np.argmin(abs(no
rthi - n_pos))]
        dSigma = np.log10(np.pi*R**2*tt*S*(dp)*DP*10**6)
        d_sigma_lateral[jdx,idx] = np.log10(np.pi*R**2*tt*S*(dp)*DP*10**
6)
        sigma_pp_lateral[jdx,idx] = a_lateral_GR[jdx,idx] - dSigma

xv, yv = np.meshgrid(np.arange(-R,R+disc,disc), np.arange(-R,R+disc,dis
c))
xv_vec = xv.flatten()
yv_vec = yv.flatten()
eq_pos = np.transpose(np.array([xv_vec,yv_vec]))
is_inside = f1.points_in_ellipse([0,0],0,R+1,R+1,eq_pos)
```

Filter Data by uncertainty

In [20]:
```
for i in range(np.shape(a_lateral)[0]):
    for j in range(np.shape(a_lateral)[1]):
        if a_lateral_uncertainy[i,j] > .04:
            a_lateral[i,j] = np.nan
            a_lateral_GR[i,j] = np.nan
            a_lateral_uncertainy[i,j] = np.nan
            sigma_pp_lateral[i,j] = np.nan
        if b_lateral_uncertainy[i,j] > .02:
            b_lateral[i,j] = np.nan
            b_lateral_GR[i,j] = np.nan
            sigma_pp_lateral[i,j] = np.nan
        if My_lateral_uncertainy[i,j] > .025:
            My_lateral[i,j] = np.nan
            My_lateral_uncertainy[i,j] = np.nan
            stress_drop_lateral[i,j] = np.nan
            #sigma_pp_lateral[i,j] = np.nan
        if My_lateral[i,j] <3.2:
            sigma_pp_lateral[i,j] = np.nan
            a_lateral[i,j] = np.nan
            a_lateral_GR[i,j] = np.nan
            a_lateral_uncertainy[i,j] = np.nan
            b_lateral[i,j] = np.nan
            b_lateral_GR[i,j] = np.nan
            My_lateral[i,j] = np.nan
            stress_drop_lateral[i,j] = np.nan
            sigma_pp_lateral[i,j] = np.nan

## Smooth results to get rid of circular artifacts
sm_base = 10
circle = np.zeros([2*sm_base,2*sm_base])
for i in range(2*sm_base):
    for j in range(2*sm_base):
        if np.sqrt((i-sm_base)**2 + (j-sm_base)**2) <= sm_base:
            circle[i,j] = 1
idx = np.where(circle==1)
a_lateral_smooth = np.zeros(np.shape(a_lateral))
a_lateral_smooth[:] = np.nan
a_lateral_smooth_GR = np.zeros(np.shape(a_lateral))
a_lateral_smooth_GR[:] = np.nan

b_lateral_smooth = np.zeros(np.shape(a_lateral))
b_lateral_smooth[:] = np.nan
b_lateral_smooth_GR = np.zeros(np.shape(a_lateral))
b_lateral_smooth_GR[:] = np.nan

My_lateral_smooth = np.zeros(np.shape(a_lateral))
My_lateral_smooth[:] = np.nan
sigma_pp_lateral_smooth = np.zeros(np.shape(a_lateral))
sigma_pp_lateral_smooth[:] = np.nan
d_sigma_lateral_smooth = np.zeros(np.shape(a_lateral))
d_sigma_lateral_smooth[:] = np.nan


for i in range(sm_base,np.shape(a_lateral)[0]-sm_base):
    for j in range(sm_base,np.shape(a_lateral)[1]-sm_base):
        a_lateral_smooth[i,j] = np.nanmean(a_lateral[idx[0][:] + i-sm_bas
e,idx[1][:] + j-sm_base])
        a_lateral_smooth_GR[i,j] = np.nanmean(a_lateral_GR[idx[0][:] + i-
sm_base,idx[1][:] + j-sm_base])
```

```
        b_lateral_smooth[i,j] = np.nanmean(b_lateral[idx[0][:] + i-sm_bas
e,idx[1][:] + j-sm_base])
        b_lateral_smooth_GR[i,j] = np.nanmean(b_lateral_GR[idx[0][:] + i-
sm_base,idx[1][:] + j-sm_base])
        My_lateral_smooth[i,j] = np.nanmean(My_lateral[idx[0][:] + i-sm_b
ase,idx[1][:] + j-sm_base])
        sigma_pp_lateral_smooth[i,j] =  np.nanmean(sigma_pp_lateral[idx
[0][:] + i-sm_base,idx[1][:] + j-sm_base])
        d_sigma_lateral_smooth[i,j] =  np.nanmean(d_sigma_lateral[idx
[0][:] + i-sm_base,idx[1][:] + j-sm_base])
```

Plot results

In [21]:
```
plt.figure(figsize=(10,9))
plt.imshow(np.flipud((b_lateral_smooth)),extent=[east_lims[0]-offset*dis
c,east_lims[1]+offset*disc,north_lims[0]-offset*disc,north_lims[1]+offset
*disc],vmin=.5,vmax=1.4)
plt.plot(field_east,field_north,'r-')
plt.xlim([230000,270000])
plt.ylim([565000,615000])
plt.colorbar()
plt.title('b-value',fontweight='bold')
plt.xlabel('Easting [m]',fontweight='bold')
plt.ylabel('Northing [m]',fontweight='bold')

plt.figure(figsize=(10,9))
plt.imshow(np.flipud((a_lateral_smooth)),extent=[east_lims[0]-offset*dis
c,east_lims[1]+offset*disc,north_lims[0]-offset*disc,north_lims[1]+offset
*disc],vmin=3.,vmax=3.8)
plt.plot(field_east,field_north,'r-')
plt.xlim([230000,270000])
plt.ylim([565000,615000])
plt.colorbar()
plt.title('a-value',fontweight='bold')
plt.xlabel('Easting [m]',fontweight='bold')
plt.ylabel('Northing [m]',fontweight='bold')

plt.figure(figsize=(10,9))
plt.imshow(np.flipud((My_lateral_smooth)),extent=[east_lims[0]-offset*dis
c,east_lims[1]+offset*disc,north_lims[0]-offset*disc,north_lims[1]+offset
*disc],vmin=3.5,vmax=4.5)
plt.plot(field_east,field_north,'r-')
plt.xlim([230000,270000])
plt.ylim([565000,615000])
plt.colorbar()
plt.title('M_Y',fontweight='bold')
plt.xlabel('Easting [m]',fontweight='bold')
plt.ylabel('Northing [m]',fontweight='bold')

plt.figure(figsize=(10,9))
plt.imshow(np.flipud((sigma_pp_lateral_smooth)),extent=[east_lims[0]-offs
et*disc,east_lims[1]+offset*disc,north_lims[0]-offset*disc,north_lims[1]+
offset*disc],vmin=-5,vmax=-4)
plt.plot(field_east,field_north,'r-')
plt.colorbar()
plt.xlim([230000,270000])
plt.ylim([565000,615000])
plt.title('Seismogenic Index',fontweight='bold')
plt.xlabel('Easting [m]',fontweight='bold')
plt.ylabel('Northing [m]',fontweight='bold')
plt.show()
```

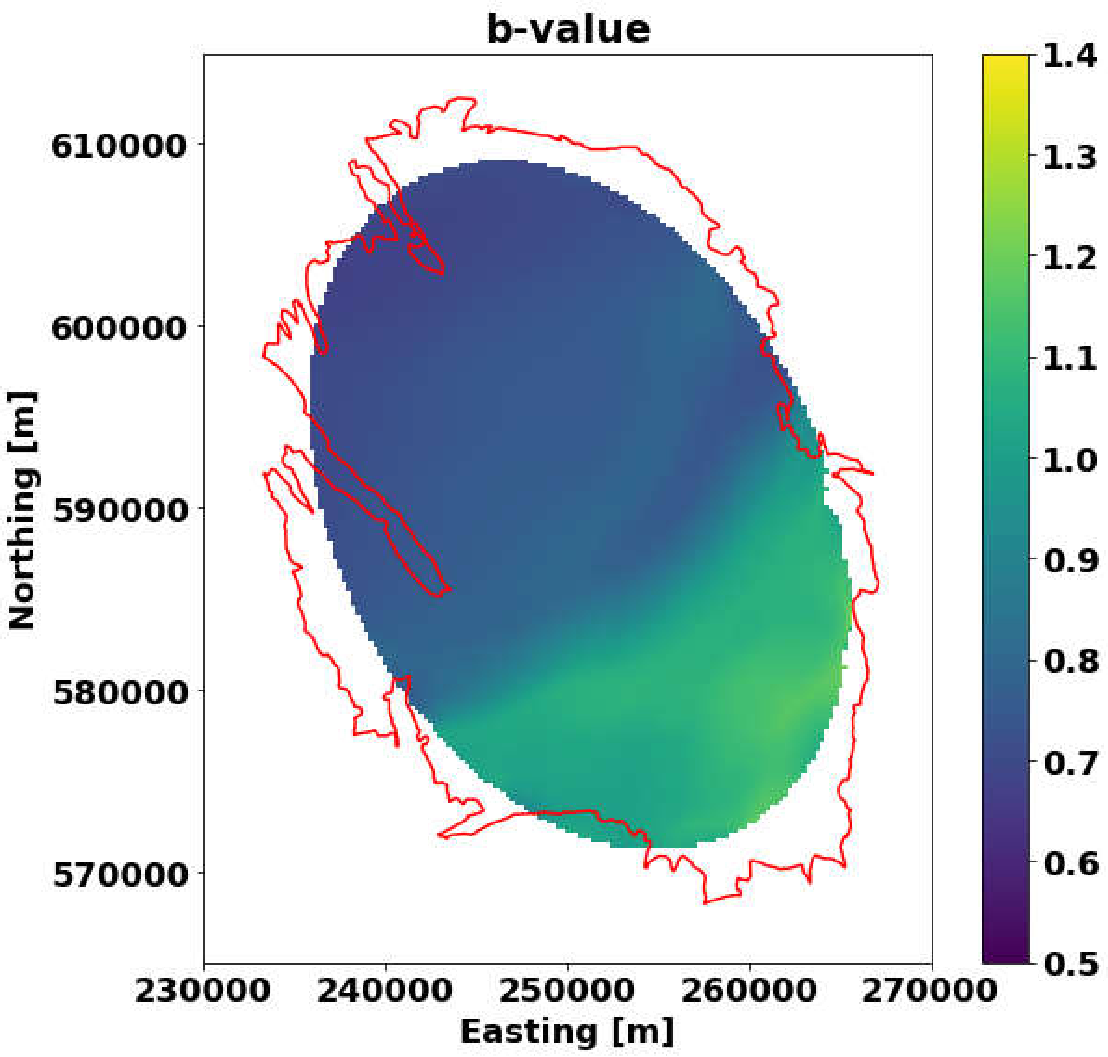
b-value
Northing [m]
610000
600000
590000
580000
570000
230000
240000
250000
260000
270000
Easting [m]
1.4
1.3
1.2
1.1
1.0
0.9
0.8
0.7
0.6
0.5

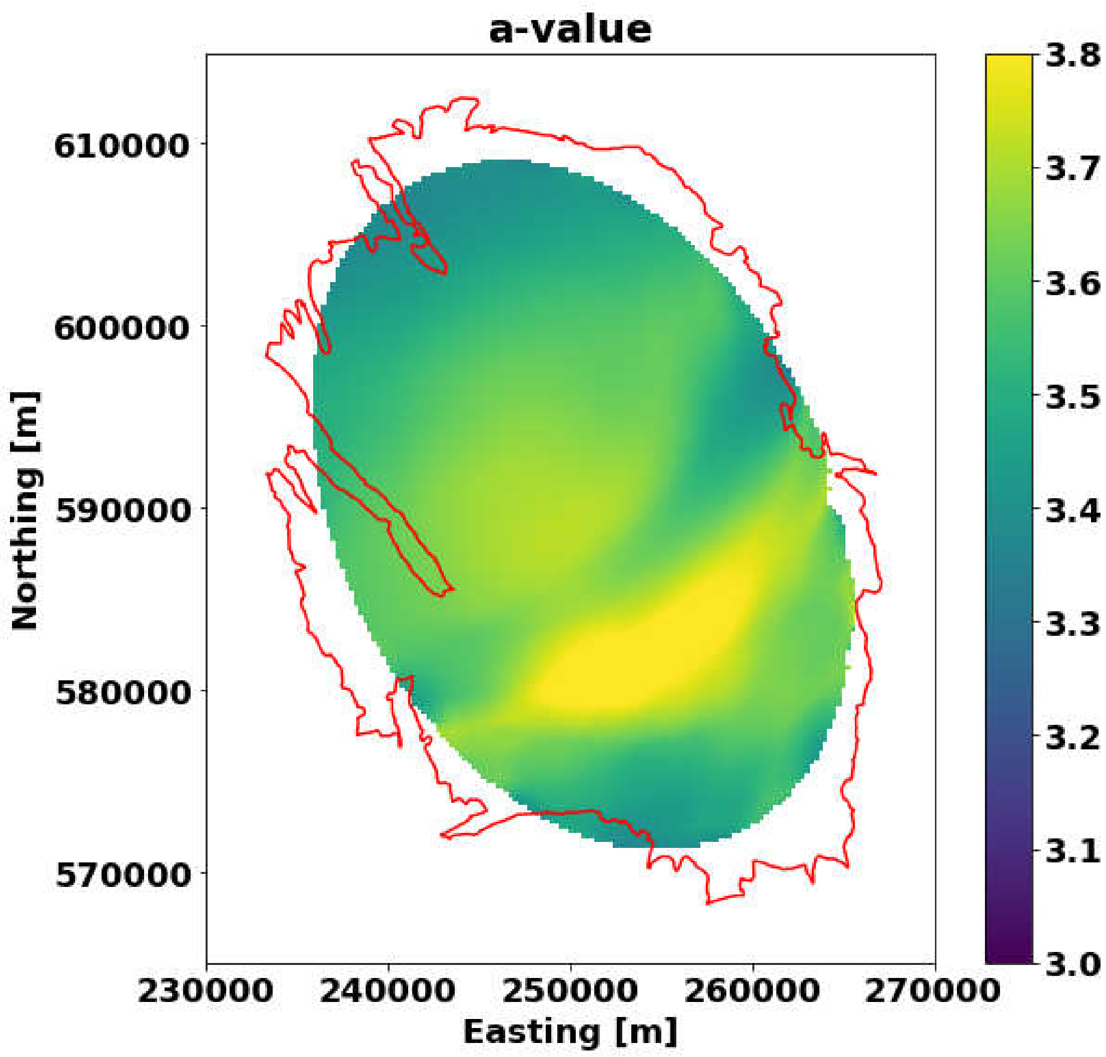
a-value
610000
600000
590000
580000
570000
Northing [m]
230000
240000
250000
260000
270000
Easting [m]
3.8
3.7
3.6
3.5
3.4
3.3
3.2
3.1
3.0

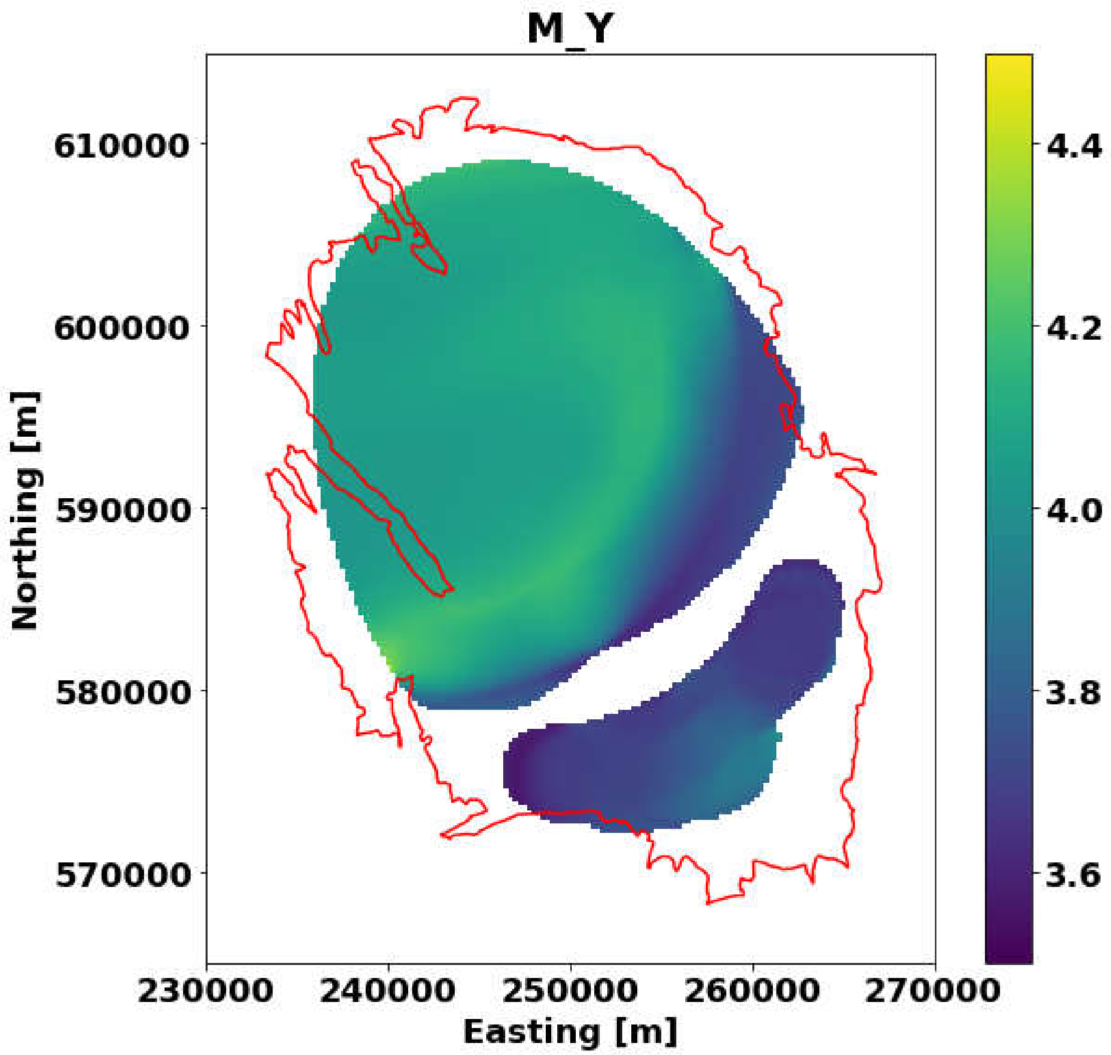
M_Y
610000
600000
590000
580000
570000
Northing [m]
230000
240000
250000
260000
270000
Easting [m]
4.4
4.2
4.0
3.8
3.6

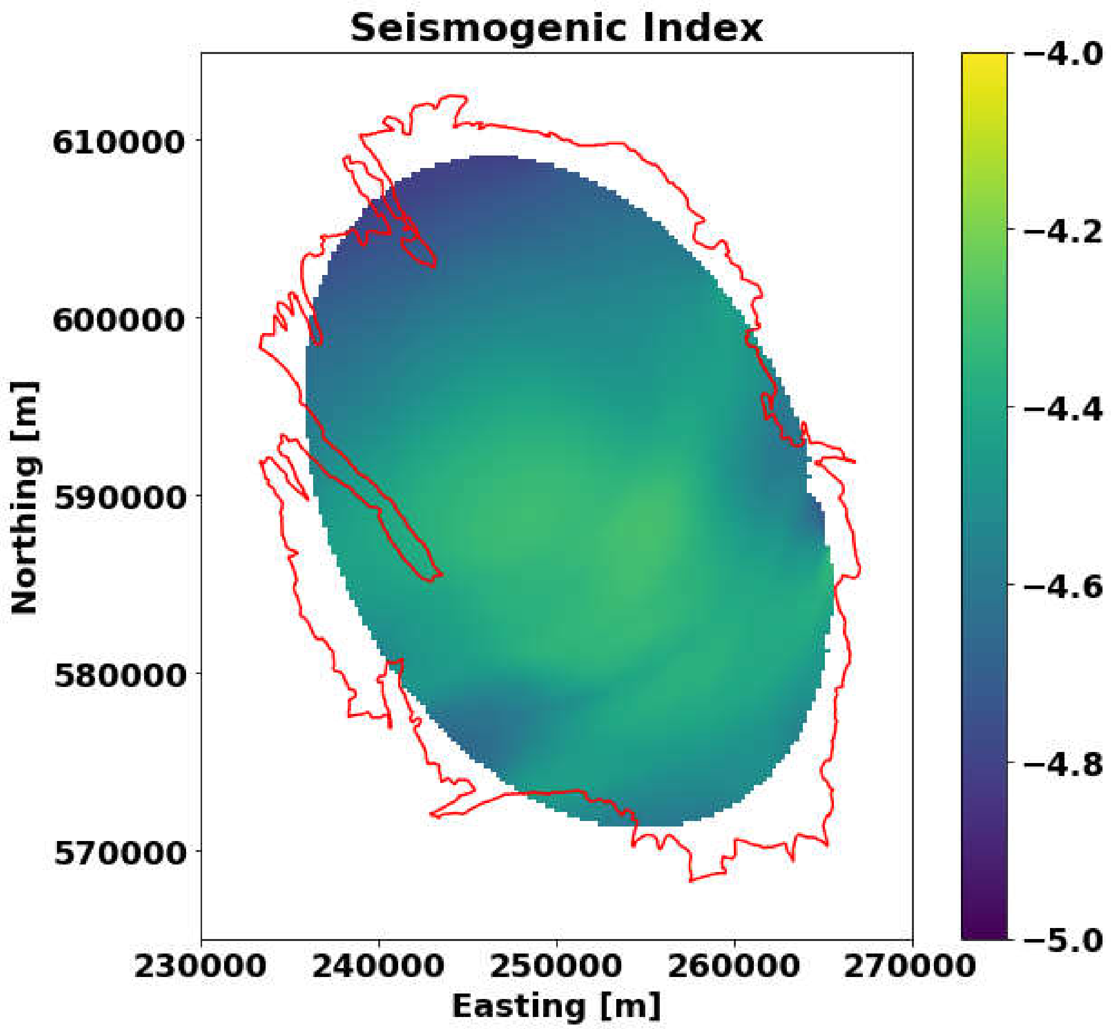
Seismogenic Index
610000
600000
590000
580000
570000
Northing [m]
230000
240000
250000
260000
270000
Easting [m]
-4.0
-4.2
-4.4
-4.6
-4.8
-5.0
